# A novel tumor suppressor protein encoded by circular AKT3 RNA inhibits glioblastoma tumorigenicity by competing with active phosphoinositide-dependent Kinase-1

**DOI:** 10.1186/s12943-019-1056-5

**Published:** 2019-08-30

**Authors:** Xin Xia, Xixi Li, Fanying Li, Xujia Wu, Maolei Zhang, Huangkai Zhou, Nunu Huang, Xuesong Yang, Feizhe Xiao, Dawei Liu, Lixuan Yang, Nu Zhang

**Affiliations:** 1 Department of Neurosurgery, The First Affiliated Hospital of Sun Yat-sen University, No 58, Zhongshan 2 Road, Guangzhou, Guangdong Province 510080 People’s Republic of China; 2 Guangdong Provincial Key Laboratory of Brain Function and Disease, Precise Medicine Institute, The First Affiliated Hospital of Sun Yat-sen University, Guangzhou, Guangdong 510080 People’s Republic of China; 3 Department of Scientific Research Section, The First Affiliated Hospital of Sun Yat-sen University, Guangzhou, Guangdong Province 510080 People’s Republic of China; 4 Department of Pathology, The First Affiliated Hospital of Sun Yat-sen University, Guangzhou, Guangdong Province 510080 People’s Republic of China

**Keywords:** circRNA, AKT3, PDK1, Glioblastoma

## Abstract

**Background:**

The RTK/PI3K/AKT pathway plays key roles in the development and progression of many cancers, including GBM. As a regulatory molecule and a potential drug target, the oncogenic role of AKT has been substantially studied. Three isoforms of AKT have been identified, including AKT1, AKT2 and AKT3, but their individual functions in GBM remain controversial. Moreover, it is not known if there are more *AKT* alternative splicing variants.

**Methods:**

High-throughput RNA sequencing and quantitative reverse transcription-PCR were used to identify the differentially expressed circRNAs in GBM samples and in paired normal tissues. High throughput RNA sequencing was used to identify circ-AKT3 regulated signaling pathways. Mass spectrometry, western blotting and immunofluorescence staining analyses were used to validate AKT3-174aa expression. The tumor suppressive role of AKT3-174aa was validated in vitro and in vivo. The competing interaction between AKT3-174aa and p-PDK1 was investigated by mass spectrometry and immunoprecipitation analyses.

**Results:**

Circ-AKT3 is a previously uncharacterized *AKT* transcript variant. Circ-AKT3 is expressed at low levels in GBM tissues compared with the expression in paired adjacent normal brain tissues. Circ-AKT3 encodes a 174 amino acid (aa) novel protein, which we named AKT3-174aa, by utilizing overlapping start-stop codons. AKT3-174aa overexpression decreased the cell proliferation, radiation resistance and in vivo tumorigenicity of GBM cells, while the knockdown of circ-AKT3 enhanced the malignant phenotypes of astrocytoma cells. AKT3-174aa competitively interacts with phosphorylated PDK1, reduces AKT-thr308 phosphorylation, and plays a negative regulatory role in modulating the PI3K/AKT signal intensity.

**Conclusions:**

Our data indicate that the impaired circRNA expression of the *AKT3* gene contributes to GBM tumorigenesis, and our data corroborate the hypothesis that restoring AKT3-174aa while inhibiting activated AKT may provide more benefits for certain GBM patients.

**Electronic supplementary material:**

The online version of this article (10.1186/s12943-019-1056-5) contains supplementary material, which is available to authorized users.

## Background

GBM is one of the most lethal human cancers. With multimodal treatments available, including surgery, radiation and chemotherapy, the median survival time of patients with GBM is only 12–14 months [1]. Although many novel strategies have been tested for years, no effective target therapy has been validated in GBM to function better than temozolomide [1, 2]. The disruption of RTK/PI3K signaling is considered to be one of the major pathways during GBM tumorigenesis and progression [3]. The aberrant activation of RTK/PI3K signaling in GBM has multiple causes: the frequent amplification/mutation of EGFR (~ 45%), the mutation/homozygous deletion of PTEN (~ 36%) and the mutation of PI3K (~ 15%); these mutations collaboratively activate this pathway in ~ 88% GBM patients [4]. AKT, a serine-threonine kinase, is a key downstream effector of the RTK/PI3K pathway. Activated AKT, which is observed in ~ 70% GBM patients, especially in those with PTEN loss [5], mediates tumor cells proliferation, survival and malignant transformation through multiple downstream targets, including GSK-3, MDM2, and mTOR et al. [6]. The above evidence suggests that AKT is a reasonable therapeutic target for some GBM patients.

The human AKT family has three isoforms, AKT1, AKT2 and AKT3. Although they share with very similar sequences, isoform-specific functions have been reported. In knockout mice, AKT1 deprivation showed a growth retardation phenotype, and mice with AKT2 deletion had type II diabetes [7, 8]. In contrast, AKT3 facilitated postnatal brain development instead of homeostasis, indicating the critical role of AKT3 in the central nervous system [9]. AKT3, but not AKT1 or AKT2, was required for the anchorage-independent growth of transformed astrocytes and human glioma cells [10]. Although AKT3 expression was reported to be downregulated in GBM, it has shown a stronger kinase activity than that of AKT2 [11]. However, AKT3 was also shown to delay GBM progression in some GBM cases, raising the controversial role of AKT3 in GBM tumorigenesis [12].

We have previously reported that circular RNA encodes functional peptides or proteins in GBM [13–15]. By using high-throughput RNA sequencing from GBM clinical samples and paired normal brain tissues, we identified that circ-AKT3 has low expression levels in GBM. Circ-AKT3 encodes a 174 amino acid novel protein, which we named AKT3-174aa. Functionally inverse to AKT3, AKT3-174aa blocks AKT thr-308 phosphorylation by competing with activated PDK1 and inhibits the proliferation, radiation resistance and tumorigenicity of GBM cells. Our results show that circ-AKT3 is a novel functional *AKT* transcript variant and that in addition to PTEN, AKT-174aa is a newly identified negative regulator of the RTK/PI3K pathway.

## Methods

### Human cancer and normal tissues

All GBM (*n* = 38 and their paired, peripheral normal brain tissues) samples were collected from the Department of Neurosurgery at the 1st Affiliated Hospital of Sun Yat-sen University. The human materials were obtained with informed consent, and the study was approved by the Clinical Research Ethics Committee.

### Animal care and ethics statement

Four-week-old female BALB/c-nu mice were purchased from the Laboratory Animal Center of Sun Yat-sen University. The mice were housed in a temperature-controlled (22 °C) and light-controlled pathogen-free animal facility with free access to food and water. All experimental protocols concerning the handling of mice were approved by the Institutional Animal Care and Use Committee of Sun Yat-sen University.

### Cell culture and RNase R treatments

All cells used in this study were tested for mycoplasma contamination and were authenticated by STR sequencing. The 293 T cell line was purchased from ATCC (ATCC number: CRL-11268), and the U373 cell line was also from ATCC (ATCC number: CRL-1620). The U251, HS683, and SW1783 cell lines were kindly provided by Dr. Suyun Huang, VCU. These cells were cultured in Dulbecco’s modified Eagle’s medium (GIBCO BRL, Grand Island, NY, USA) supplemented with 10% fetal bovine serum (GIBCO BRL, Grand Island, NY, USA) according to standard protocols. GICs were kindly provided by Dr. Jeremy Rich, UCSD. These cells were cultured in DMEM/F12 medium supplemented with B27 supplement (Life Technologies), bFGF and EGF (20 ng/ml each). iPS derived neural stem cells were kindly provided by Dr. Peng Xiang, SYSU. These cells were culture with StemPro® NSC SFM (Cat. no. A10509–01) supplemented with 2 mM GlutaMAX™-I Supplement (Cat. no. 35050), 6 U/mL heparin (Sigma, Cat. no. H3149), and 200 μM ascorbic acid (Sigma, Cat. no. A8960). NHA were purchased from Lonza and were cultured using an AGMTM Astrocyte Growth Medium Bullet Kit™ (Lonza, Walkersville, MD, USA) as recommended by the manufacturer. RNase R (Epicenter Biotechnologies, Madison, WI, USA) treatment (20 U/μl) was performed on total RNA (20 μg) at 37 °C for 15 min.

### RNA sequencing

Total RNA was extracted using Trizol reagent kit (Invitrogen, Carlsbad, CA, USA) according to the manufacturer’s protocol. RNA quality was assessed on an Agilent 2100 Bioanalyzer (Agilent Technologies, Palo Alto, CA, USA) and checked using RNase free agarose gel electrophoresis. After total RNA was extracted, eukaryotic mRNA was enriched by Oligo(dT) beads, while prokaryotic mRNA was enriched by removing rRNA by Ribo-ZeroTM Magnetic Kit (Epicentre, Madison, WI, USA). Then the enriched mRNA was fragmented into short fragments using fragmentation buffer and reversely transcripted into cDNA with random primers. Second-strand cDNA were synthesized by DNA polymerase I, RNase H, dNTP and buffer. Then the cDNA fragments were purified with QiaQuick PCR extraction kit (Qiagen, Venlo, The Netherlands), end repaired, poly(A) added, and ligated to Illumina sequencing adapters. The ligation products were size selected by agarose gel electrophoresis, PCR amplified, and sequenced using Illumina HiSeq2500 by Gene Denovo Biotechnology Co. (Guangzhou, China).

Data was mapped to reference genome by TopHat2 (version 2.1.1), then transcripts abundances were quantified by software RSEM (version 1.2.19). Firstly, a set of reference transcript sequences were generated and preprocessed according to transcripts (in FASTA format) and gene annotation files (in GTF format). Secondly, reads were realigned to the reference transcripts by Bowtie alignment program and the resulting alignments were used to estimate transcript abundances. The transcript expression level was normalized by using FPKM (Fragments Per Kilobase of transcript per Million mapped reads). Value of transcripts from the same gene were merged to obtain reads counts and expression level at gene level. Differentially expressed genes (DEGs) were also identified by the edgeR package (version 3.12.1) (http://www.r-project.org/) with general linear model and a threshold of fold change > 2 and FDR < 0.05. KEGG pathway enrichment analysis (Fisher’s Exact Test) was performed for DEGs.

### Antibodies

Antibodies against pan-AKT (#4691, 1:1000), phospho-AKT Thr308 (#13038, 1:1000), phospho-AKT Ser473 (#4060, 1:1000), AKT1 (#2938, 1:1000), AKT2 (#3063, 1:1000), AKT3 (#14982, 1:1000), PDK1 (#13037, 1:1000) and p-PDK1 (#3438, 1:1000) were from Cell Signaling Technology (Danvers, MA, USA). Antibodies against γ- H2AX (ab2893; 1:1000), PTEN (ab32199; 1:10000), EGFR (ab32430; 1:5000) were from Abcam (Cambridge, MA, USA). Antibodies against flag (F1804, 1 mg/mL; 1:1000) and beta-tubulin (T5201; 1:5000) were from Sigma-Aldrich (St. Louis, MO, USA).

### Statistical analysis

Statistical tests were conducted using GraphPad Prism (Version 8; La Jolla, CA, USA) software unless otherwise indicated. The data are presented as the mean ± s.e.m. from three independent experiments. For the parametric data, unpaired, two-tailed Student’s t-tests were used. For nonparametric data, the two-sided Mann–Whitney test was used. Data distribution was assumed to be normal, but this was not formally tested. A level of *P* < 0.05 was used as the cutoff for significant differences. For all experiments, analyses were done in biological triplicates. No animals or data points were excluded from the analyses for any reason. Blinding and randomization were performed in all experiments. Statistical analyses for the RNA-seq data are described above in the respective sections.

Full material and methods were described in Table 1 and Additional file 1.

**Table 1:** Primers and oligos used

RT-PCR primers			
	Forward primer(5‘to 3’)	Reverse primer(5‘to 3’)	Amplified product(bps)
QPCR Circ-AKT3(Divergent primers)	AAGTGGCACACACTCTAACTG	GTTTTCATTAACTGGCATTCTCG	150
QPCR Linear-AKT3(Convergent primers)	GGAGTCATCATGAGCGATGT	TAACTGGCATTTTGCCACTG	192
QPCR-AKT1	CATGAGCGACGTGGCTATTG	GCCTCACGTTGGTCCACATC	150
QPCR-AKT2	AAGAAGGCTGGCTCCACAAG	GCATTCTGCTACGGAGAAGT	158
QPCR-beta-actin	ACAGAGCCTCGCCTTTGCCGAT	CTTGCACATGCCGGAGCCGTT	109
Plasmid construction primers			
OV-circ-AKT3	TTCGAATTCAGTGCTGAGATTACAGGCGTGAG	TTCGAATTCAGTGCTGAGATTACAGGCGTGAG	686
OV-AKT3-174aa-flag	TTCGAATTCATGAAAACAGAACGACCAAAGC	AATGGATCCTTACTTGTCATCGTCATCCTT	609
Rluc-PCR	AGGCTAGCGCCACCATGGCTTCCAAGGTGT	TTATTACTGCTCGTTCTTCAGCAC	953
luc-PCR	GCCACCATGGCCGATGCTAA	CGCTCGAGTTACACGGCGATCTTGCCGCCTT	1667
Circ-AKT3-IRES-WT	ATGGTACCAATGGACAGAAGCTATCCAGGCTGTA	CTGGAATTCCCTTCTCTCGAACCAAAATAACT	233
Circ-AKT3-IRES-Del-1	ATGGTACCAATGGACAGAAGCTATCCAGGCTGTA	CTGGAATTCATCTCTTCCTCTCCTATATTATCAA	126
Circ-AKT3-IRES-Del-2	ATGGTACCTGGATGCCTCTACAACCCATCA	CTGGAATTCCCTTCTCTCGAACCAAAATAAC	125
Probes			
Circ-AKT3-FISH-probe	Cy3-TTTCATTAACTGGCATTCTCGCCCCCATTAAC		
Northern-Circ-AKT3-probe	TTTCATTAACTGGCATTCTCGCCCCCATTAAC-DIG		
Northern-U6-probe	TCTTCTCTGTATCGTTCCAATTTTAGTATATGTGC-DIG		
siRNA sequences			
Name	sense(5'-3')	antisense(5'-3')	
Circ-AKT3-siRNA-1	GGGGCGAGAAUGCCAGUUAAUtt	AUUAACUGGCAUUCUCGCCCCtt	
Circ-AKT3-siRNA-2	GGCGAGAAUGCCAGUUAAUGAtt	UCAUUAACUGGCAUUCUCGCCtt	
Circ-AKT3-siRNA-NC	GCGCCCUGAUUGCCUGAAAUAtt	UAUUUCAGGCAAUCAGGGCGCtt	

## Results

### Circ-AKT3 has low expression levels in GBM samples/cell lines compared with its expression in normal brain tissues/normal human astrocytes (NHA) and neural stem cells

**Fig. 1:**
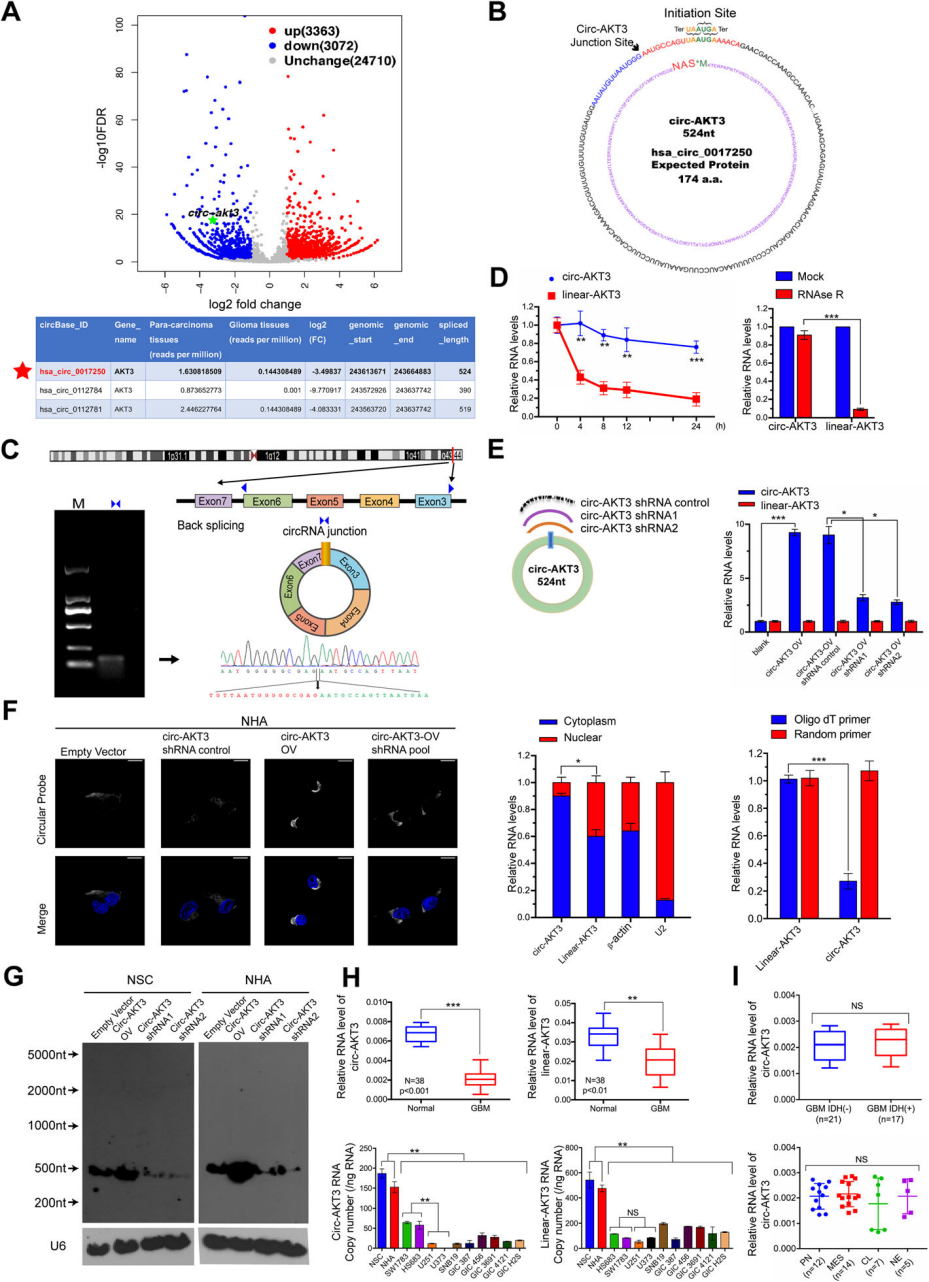
Identification of circ-AKT3 as a novel *AKT* gene alternative splicing transcript. **a** Upper, Volcano plot of circRNA expression. X-axis: log2 ratio of circRNA expression levels between normal and tumor tissues. Y-axis: the FDR value (−log10 transformed) of circRNAs. The green dot indicates hsa_circ_0017250 (circ-AKT3). Lower, identified three circRNAs from *AKT* gene in our RNA-seq. **b** Illustration of the complete ORF in circ-AKT3. Circ-AKT3 used an overlap start-stop codon UAAUGA. **c** Illustration of the annotated genomic region of AKT3, the putative different mRNA splicing forms (linear splicing and ‘head-to-tail’ splicing) and the validation strategy for the circular exon 3–7 (circ-AKT3). Sanger sequencing following PCR conducted using the indicated divergent flanking primers showed the ‘head-to-tail’ splicing of circ-AKT3 in HEK293T cells. **d** Left, relative RNA level of circ-AKT3 and linear AKT3 in different time point. Right, relative RNA level of circ-AKT3 and linear AKT3 treated with RNase R. Error bars represent three independent experiments, **, *p* < 0.01, ***, *p* < 0.001. **e** Left, circ-AKT3 overexpression plasmid vector (not shown) and two circ-AKT3 junction shRNAs and a control shRNA were designed, transfected into HEK293T cells. Right, relative circ-AKT3 and linear AKT3 RNA level were decided by q-PCR. **f** Left, Fluorescence in situ hybridization (FISH) with junction-specific probes were used to decide the localization of circ-AKT3. Circ-AKT3 overexpression or shRNA were used independently or in combination to show the specificity of these probes. Scale bars, 20 μM. Middle, cytoplasmic and nuclear fractions were isolated, circ-AKT3 and linear AKT3 expression were decided. β-actin and U2 RNA served as cytoplasmic and nuclear RNA markers. Right, total RNA from HEK293T cells were reversely transcript with Oligo dT primers or random primers, and circ-AKT3 or linear AKT3 mRNA were decided by q-PCR. Error bars represent three independent experiments, *, *p* < 0.05, ***, *p* < 0.001. **g** Northern blots using the junction-specific circular probe were used to detect circ-AKT3 in circ-AKT3 overexpressed or plus circ-AKT3 shRNA transfected NSC and NHA cells. **h** Relative circ-AKT3 and linear AKT3 RNA level of GBM clinical samples and paired adjacent normal tissues in a cohort of 38 GBM patients, or of NSC, NHA and GBM cell lines. Error bars represent three independent experiments, ***, *p* < 0.001. **i** Circ-AKT3 RNA level and its correlation with IDH1 status or molecular subtypes. Error bars represent three independent experiments

We previously generated a circRNA profiling database using the RNA-seq data of ribosomal RNA-depleted total RNA from clinical GBM tissues and paired adjacent normal tissues [13]. The RNA from ten GBM samples was combined for the tumor group, and the RNA from their periphery normal tissues was combined as the normal group. Both tumor group and normal group were sequenced on an Illumina HiSeq™ 2500 with RNase R enrichment. The reads obtained were mapped to reference ribosomal RNA sequences (Bowtie2, http://bowtie-bio.sourceforge.net/bowtie2/) and to a reference genome (TopHat2, http://ccb.jhu.edu/software/tophat/) [16, 17]; 20-mers from both ends of the unmapped reads were extracted and aligned to the reference genome to find unique anchor positions within the splice site. Anchor reads that aligned in the reverse orientation (head-to-tail) indicated circRNA splicing and were then subjected to find_circ (https://omictools.com/find-circ-tool) to identify the circRNAs [18]. A candidate circRNA was called if it was supported by at least two unique back-spliced reads in one sample. A total of 31,145 circRNAs were identified by this approach (PRJNA355185 (SRP095744)), 6442 of which were matched in circBase (http://www.circbase.org/) [19]. By analyzing the most differentially expressed circRNAs between tumor group and normal group, we identified circ-AKT3 (Fig. 1a, upper panel). There are three circular RNAs generated from the *AKT3* gene (hsa_circ_0017250, hsa_circ_0112784 and hsa_circ_0112781) that were detected in our RNA-seq data, and all of them had a lower expression in GBM compared with that in normal tissues (Fig. 1a, lower panel). Another circRNA generated from *AKT3* (hsa_circ_0000199) was recently reported to be upregulated in gastric cancer [20]. We did not find it in our RNA-seq data, which could be explained by organ-specificity. Of these three circRNAs, hsa_circ_0017250 and hsa_circ_0112781 were more abundantly expressed in normal brain tissues. Moreover, only hsa_circ_0017250 contains a complete ORF as shown in Fig. 1b. Due to the significant lower level of hsa_circ_0017250 (termed as circ-AKT3) in tumor group compare with that in normal group (11 folds), we next explored its endogenous expression.

Circ-AKT3 is generated from exon 3 to exon 7 of the *AKT3* gene with a full length of 524 nt. Divergent primers spanning the circ-AKT3 junction amplified the predicted PCR products, and Sanger sequencing confirmed the circular junction (Fig. 1c). Compared with linear form of *AKT3* mRNA, circ-AKT3 is resistant to RNase R digestion [21] and has a longer half-life (Fig. 1d). We designed a circ-AKT3 overexpression plasmid and two circ-AKT3 junction shRNAs (Fig. 1e, upper panel). These plasmids or shRNAs could specifically upregulate or knockdown the circ-AKT3 level in 293 T cells, without affecting the linear AKT3 mRNA (Fig. 1e, lower panel). Immunofluorescence was performed using a junction-specific probe and showed that circ-AKT3 mostly localized in the cytoplasm of NHA cells and that the overexpression or knockdown of circ-AKT3 could enhance or attenuate the fluorescence signal, suggested the specificity of the probes (Fig. 1f, left panel). Cell fraction qPCR using circ-AKT3 or linear circ-AKT3-specific primers further confirmed the cytoplasmic localization of this circRNA. Moreover, compare with random primers reversely transcript cDNA, oligo dT reversely transcript cDNA could not amplify circ-AKT3 (Fig. 1f, right panel). Northern blot analysis verified the ~ 524 nt band of endogenous circ-AKT3 in NSC and NHA cells, which could be enhanced or attenuated by the overexpression or stable knockdown of circ-AKT3, respectively, by the indicated constructions (Fig. 1g). By using junction-specific primers, we explored circ-AKT3 expression patterns in several established GBM cell lines, glioblastoma-initiating cells (GICs) and a cohort of 38 GBM patient tissues as we previously reported [13]. Compared with those in NSC and NHA cells, GBM cells and GICs expressed lower levels of circ-AKT3. In clinical samples, circ-AKT3 expression was also low in GBM samples compared with that in normal brain tissues (Fig. 1h, left). We also found that linear *AKT3* mRNA had a lower expression in GBM cells or clinical samples than the expression in normal brain tissues by using AKT3 mRNA-specific primers (Figs. 1h, right) as previously reported [11]. We did not find that the circ-AKT3 expression had a significant correlation with the GBM molecular subtypes or the IDH1 status (Fig. 1i).

### Circ-AKT3 encodes a 174 amino acid (aa) novel protein, AKT3-174aa

**Fig. 2:**
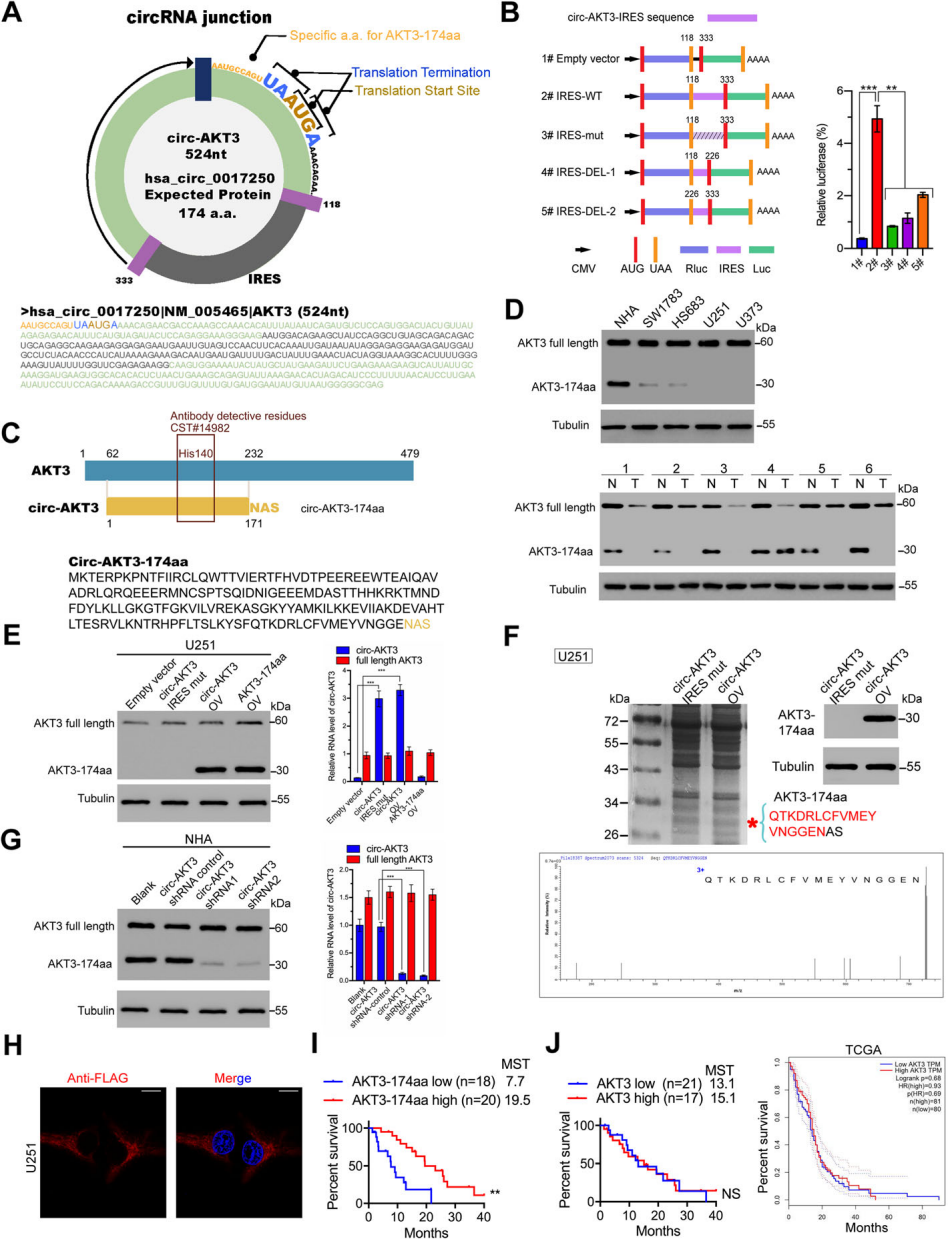
Circ-AKT3 encodes a 174aa novel protein termed as AKT3-174aa. **a** Upper panel, the putative ORF in circ-AKT3. Lower panel, the sequences of the putative ORF are shown. Note that the circ-AKT3 used an overlap start-stop codon. **b** The putative IRES activity in circ-AKT3 was tested. Upper panel, IRES sequences in circ-AKT3 or its different truncations/mutation were cloned between Rluc and Luc reporter genes with independent start and stop codons. Lower panel, the relative luciferase activity of Luc/Rluc in the above vectors was tested. Error bars represent three independent experiments, **, *p* < 0.01, ***, *p* < 0.001. **c** Illustration of AKT3-174aa sequence and AKT3 sequence. The antibody used in the study recognized both proteins. **d** AKT3 and AKT3-174aa expression were detected in established cell lines and several paired GBM samples. **e** U251 cells were transfected with empty vector, circ-AKT3 IRES mutated vector, circ-AKT3 vector and linearized AKT3 vector, respectively. Circ-AKT3, AKT3 and AKT3-174aa level were decided. Error bars represent three independent experiments, ***, *p* < 0.001. **f** Total proteins from circ-AKT3 or control plasmid-transfected U251 cells were separated via SDS-PAGE. AKT3-174aa overexpression was confirmed by immunoblotting. The differential gel bands between 26 kD and 34 kD was cut and subjected to LC-MS/MS. The identified AKT3-174aa amino acids are shown in red. **g** NHA cells were transfected with control shRNA or circ-AKT3 shRNAs. Circ-AKT3, AKT3 and AKT3-174aa level were decided. Error bars represent three independent experiments, ***, *p* < 0.001. **h** Flag tagged AKT3-174aa was transfected into U251 cells. Immunofluorescence staining using anti-Flag was performed to show the AKT3-174aa cellular localization. Scale bars, 20 μM. **i** Semi-quantitative analysis of AKT3-174aa expression level and GBM patient overall survival (OS) in the 38 patient cohort. **, *p* < 0.01. **j** Left, semi-quantitative analysis of AKT3 expression level and GBM patient overall survival (OS) in the 38 patient cohort. Right, semi-quantitative analysis of AKT3 expression level and GBM patient overall survival (OS) in TCGA data base

Circ-AKT3 has a predicted ORF, which may encode a 174 aa protein by using an overlapping start-stop codon ‘UAAUGA’ (Fig. 2a). We verified the activity of the predicted IRES in circ-AKT3 by using a dual-luciferase assay, as shown in Fig. 2b. Driven by this IRES, the 174 aa product covers the same sequences as AKT3 from amino acids 62–232, with a special C-terminal of ‘Ans Ala Ser’. We used an antibody against the middle part of AKT3, which should recognize both AKT3 and AKT3-174aa (Fig. 2c). We found that in established cell lines and paired clinical GBM samples, AKT3-174aa was lowly expressed in cancerous cells/tissues compared with that in normal cells/tissues (Fig. 2d and Additional file 2: Figure S1). To confirm that the 174 aa predicted protein was encoded by circ-AKT3, we used the circ-AKT3 overexpression plasmid, described in Fig. 1, and a linearized AKT3-174aa overexpression plasmid. In U251 cells, which has lower endogenous circ-AKT3 levels, the transfection circ-AKT3 and the linearized AKT-174aa plasmid both resulted in the predicted AKT-174aa band, while the overexpression circ-AKT3 IRES-Mut plasmid did not (Fig. 2e). Mass spectrometry followed by SDS-PAGE using circ-AKT3 overexpression U251 cells further confirmed the AKT3-174aa peptide sequences in the predicted molecular weight (Fig. 2f). In contrast, two junction-specific shRNAs targeting circ-AKT3 could reduce AKT3-174aa expression in NHA cells, which have high endogenous levels of circ-AKT3, without affecting the linear AKT3 mRNA (Fig. 2g). These results prove that circ-AKT3 encoded this 174 aa novel protein, which we termed AKT3-174aa. Immunofluorescence using an anti-flag antibody confirmed the cytoplasmic localization of AKT3-174aa-Flag in U251 cells, as shown in Fig. 2h. In clinical samples, AKT3-174aa expression negatively correlated with GBM patient prognosis (the cohort of 38 GBM patients, determined by semiquantitative immunoblotting as we previously described [14]); this suggests that AKT3-174aa may be a prognostic marker for GBM patients (Fig. 2i). In contrast, the linear AKT3 expression levels did not have a correlation with the overall survival of GBM patients in this study cohort or in the TCGA database (Fig. 2j).

### AKT3-174aa as a tumor suppressor that inhibits the GBM malignant phenotype

**Fig. 3:**
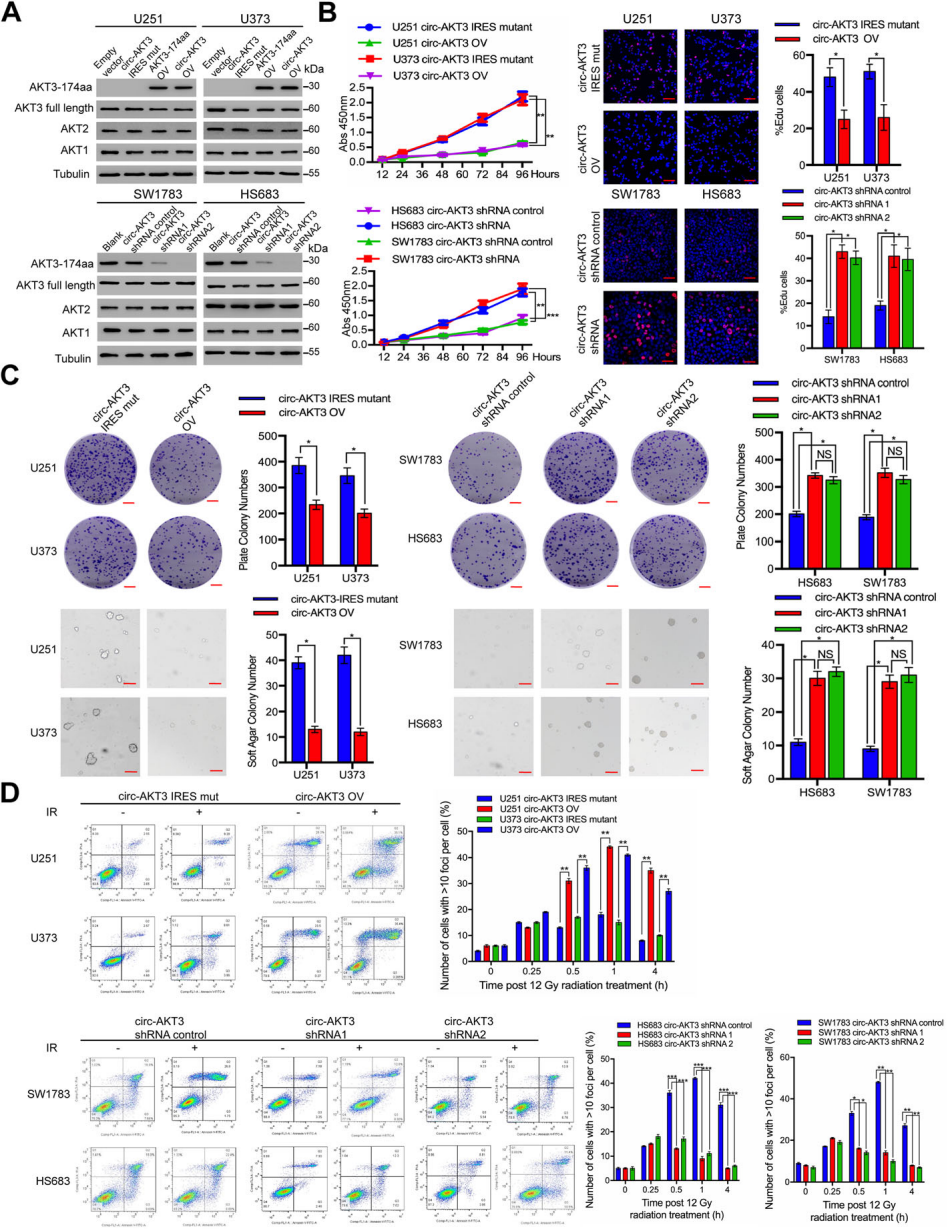
Tumor suppressive functions of AKT3-174aa in glioma cell lines. **a** U251, U373 glioma cells were transfected with circ-AKT3, linearized AKT3-174aa and control vectors; SW1783, Hs683 glioma cells were transfected with circ-AKT3 shRNA or control shRNA. AKT1, AKT2, AKT3 and AKT3-174aa level were determined by immunoblot. **b** Upper, cell proliferation, EdU incorporation of circ-AKT3, linearized AKT3-174aa transfected U251 and U373 cells and their control cells were decided. Lower, cell proliferation, EdU incorporation of circ-AKT3 shRNA transfected SW1783 and Hs683 cells and their control cells were decided. Error bars represent three independent experiments, *, *p* < 0.05, **, *p* < 0.01, ***, *p* < 0.001. **c** Left, plate colony formation and anchorage independent growth of circ-AKT3, linearized AKT3-174aa transfected U251 and U373 cells and their control cells were decided. Right, plate colony formation and anchorage independent growth of circ-AKT3 shRNA transfected SW1783 and Hs683 cells and their control cells were decided. Error bars represent three independent experiments, *, *p* < 0.05.**d** Upper, radiation resistance (cell cytometry or H2AX foci counting followed by 12Gy radiation) of circ-AKT3, linearized AKT3-174aa transfected U251 and U373 cells and their control cells were decided. Lower, radiation resistance (cell cytometry followed by 12Gy radiation) of circ-AKT3 shRNA transfected SW1783 and Hs683 cells and their control cells were decided. Error bars represent three independent experiments, *, *p* < 0.05

To explore the biological functions, we established two AKT3-174aa overexpression cell lines and two AKT3-174aa stable knockdown cells, according their endogenous circ-AKT3 expression level, by using a circ-AKT3 overexpression plasmid, a linearized AKT3-174aa plasmid or a circ-AKT3 junction shRNA expression plasmid. The IRES-mutated circ-AKT3 and the scrambled shRNA were used as negative controls (Additional file 2: Figure S2). Overexpressed circ-AKT3 or linearized AKT3-174aa, but not IRES-mutated circ-AKT3, elevated the AKT3-174aa level in U251 and U373 cells without affecting the AKT1, AKT2 or AKT3 expression. The stable knockdown of circ-AKT3 in SW1783 cells and in Hs683 anaplastic glioma cells only reduced AKT3-174aa expression, instead of reducing that of AKT1, AKT2 and AKT3 (Fig. 3a). The overexpression of circ-AKT3, but not of IRES-mutated circ-AKT3, inhibited cell proliferation in both U251 and U373 cells, while knocking down circ-AKT3 promoted SW1783 and Hs683 cell growth (Fig. 3b). The AKT3-174aa expression level negatively regulated plate colony formation and anchorage-independent growth (Fig. 3c). Additionally, AKT3-174aa expression enhanced the GBM cell sensitivity to radiation while AKT3-174aa knockdown strengthened the insensitivity of anaplastic astrocytoma cells to IR, supporting the tumor-suppressive role of AKT3-174aa (Fig. 3d).

### AKT3-174aa, but not circ-AKT3, exerts the biological functions

**Fig. 4:**
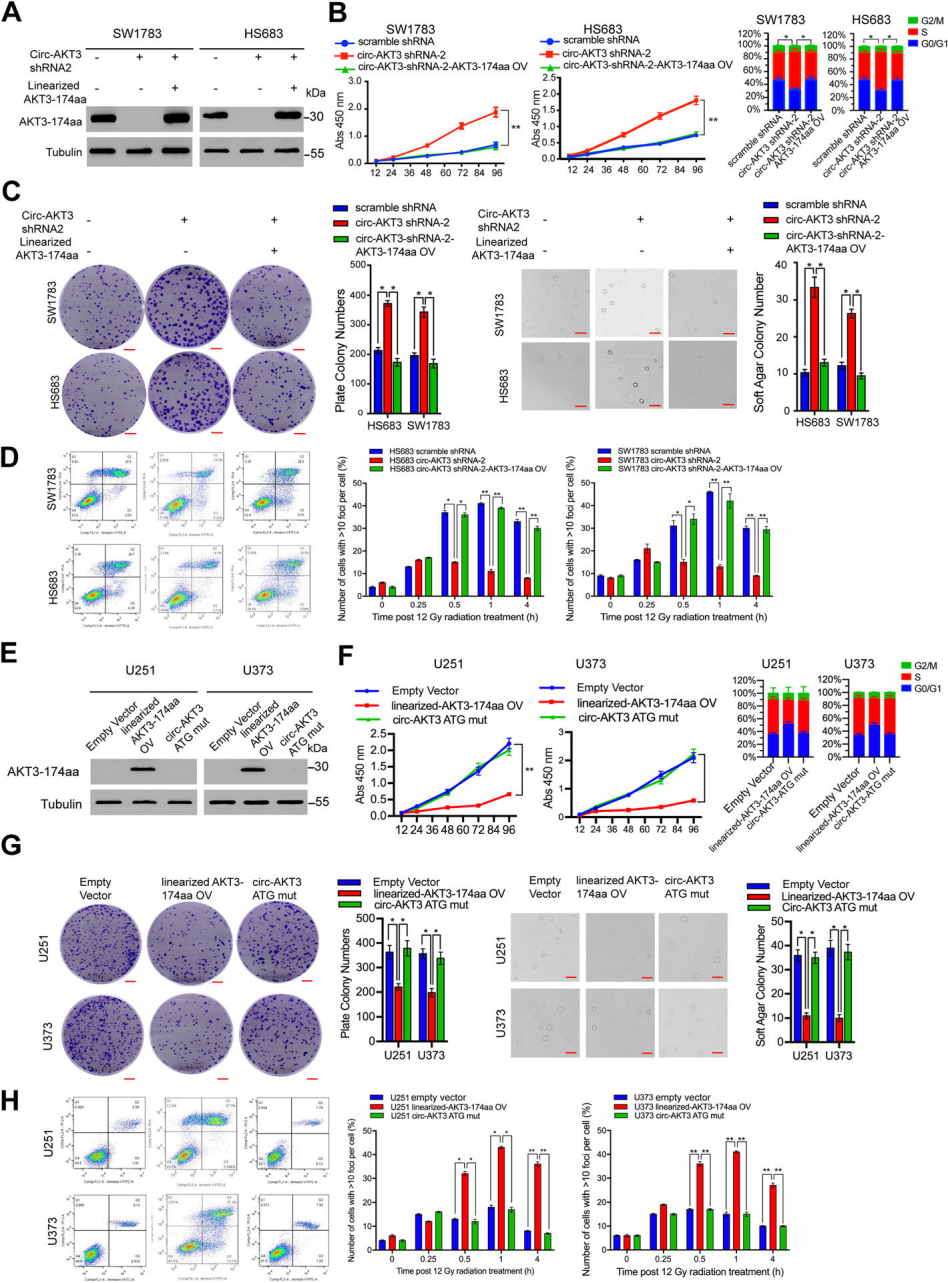
AKT3-174aa, but not circ-AKT3, exerts the biological function. **a** SW1783 and Hs683 cells were transfected with circ-AKT3 shRNA or circ-AKT3 shRNA plus linearized AKT3-174aa overexpression plasmid. AKT3-174aa expression was decided by immunoblot. **b** Cell proliferation and cell cycle analysis of SW1783 and Hs683 expressing circ-AKT3 shRNA, circ-AKT3 shRNA plus linearized AKT3-174aa and their control cells. Error bars represent three independent experiments, *, *p* < 0.05, **, *p* < 0.01, ***, *p* < 0.001. **c** Plate colony formation and anchorage independent growth of SW1783 and Hs683 expressing circ-AKT3 shRNA, circ-AKT3 shRNA plus linearized AKT3-174aa and their control cells. Error bars represent three independent experiments, *, *p* < 0.05. **d** Radiation resistance (cell cytometry or H2AX foci counting followed by 12Gy radiation) of SW1783 and Hs683 expressing circ-AKT3 shRNA, circ-AKT3 shRNA plus linearized AKT3-174aa and their control cells. Error bars represent three independent experiments, *, *p* < 0.05. **e** U251 and U373 cells were transfected with linearized AKT3-174aa plasmid or ATG mutated circ-AKT3 plasmid, and AKT3-174aa was determined by immunoblot. **f** Cell proliferation and cell cycle analysis of U251 and U373 cells transfected with linearized AKT3-174aa plasmid or ATG mutated circ-AKT3 plasmid. Error bars represent three independent experiments, *, *p* < 0.05, **, *p* < 0.01. **g** Plate colony formation and anchorage independent growth of U251 and U373 cells transfected with linearized AKT3-174aa plasmid or ATG mutated circ-AKT3 plasmid. Error bars represent three independent experiments, *, *p* < 0.05. **h** Radiation resistance (cell cytometry or H2AX foci counting followed by 12Gy radiation) of U251 and U373 cells transfected with linearized AKT3-174aa plasmid or ATG mutated circ-AKT3 plasmid. Error bars represent three independent experiments, *, *p* < 0.05, **, *p* < 0.01

Although the IRES-mutated circ-AKT3 plasmid could not translate AKT3-174aa in vivo, the mutated circRNA structure may affect its potential functions. To further exclude the possibility that circ-AKT3, but not AKT3-174aa, induced the above functions, we restored AKT3-174aa expression in SW1783 and Hs683 cells with the stable knockdown of circ-AKT3 by using an AKT3-174aa linearized plasmid (Fig. 4a). AKT3-174aa re-expression reversed the malignant phenotypes induced by the stable knockdown of circ-AKT3 (Fig. 4b-d). In contrast, the overexpression circ-AKT3 ATG-mutated plasmid (mutated at the start codon of circ-AKT3), which merely alter circ-AKT3 expression and structure, could not inhibit the malignant phenotype of the U251 and U373 cells, including cell proliferation, plate and anchorage-independent colony formation, and IR resistance (Fig. 4e-h). The above results prove that AKT3-174aa, but not circ-AKT3, exerted a tumor-suppressive role in GBM.

### AKT3-174aa inhibits the phosphorylation of AKT Thr308

**Fig. 5:**
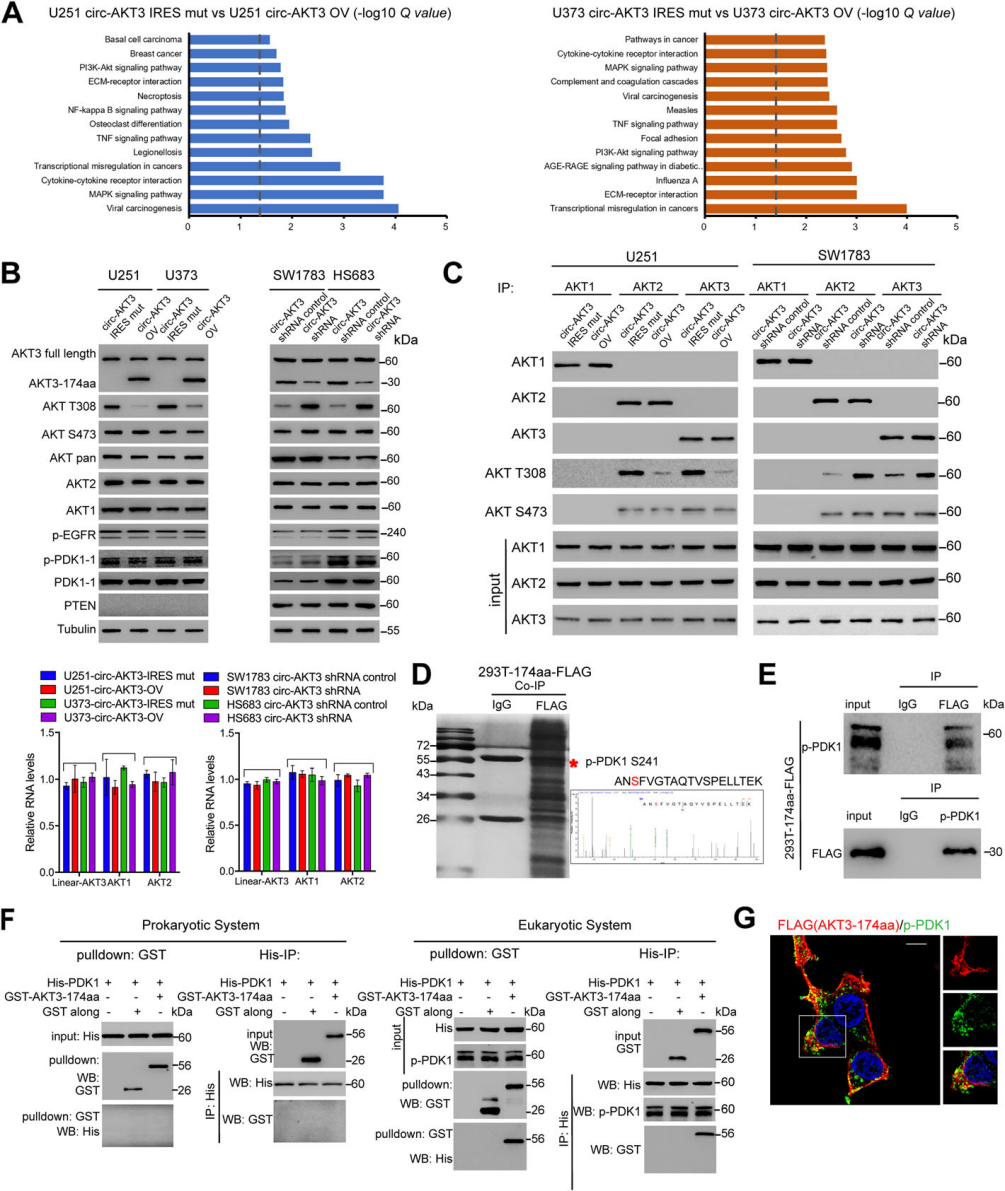
AKT3-174aa interacts with p-PDK1 and prevents AKT thr308 phosphorylation. **a** RNA sequencing was conducted in U251-circ-AKT3 IRES mut, U251-circ-AKT3, U373-circ-AKT3 IRES mut, U373-circ-AKT3 cells. Kyoto Encyclopedia of Genes and Genomes (KEGG) pathway analysis was performed to find the most differentially changed signaling pathways. **b** Upper, U251, U373, SW1783 and Hs683 cells were overexpressed or knocked down circ-AKT3, according to their endogenous AKT3-174aa expression level. The AKT3-174aa, AKT1, AKT2, AKT3, AKT-pan, AKT-thr308, AKT-ser473, p-EGFR, PTEN level were determined. Lower, AKT1, AKT2 and AKT3 mRNA level were determined in above cells, respectively. **c** IP assay using AKT1, AKT2 and AKT3 antibodies were performed in indicated cells followed by immunoblot using AKT1, AKT2, AKT3, AKT-thr308, AKT-ser473 antibodies. **d** Total proteins from Flag-AKT3-174aa plasmid-transfected HEK293T cells were separated via SDS-PAGE. P-PDK1 was identified by LC/LC-MS in AKT3-174aa protein complex. **e** Mutual interaction of p-PDK1 and Flag-AKT3-174aa were determined by IP. **f** Left, prokaryotic purified His-tagged-PDK1, GST-tagged-AKT3-174aa and GST was subjected to GST pull down or His-IP. Right, eukaryotic purified His-tagged-PDK1, prokaryotic purified GST-tagged-AKT3-174aa and GST was subjected to GST pull down or His-IP. **g** Flag-tagged AKT3-174aa was transfected into U251 cells and immunofluorescence was performed using anti-Flag and anti-p-PDK1 antibody. Scale bar, 20 μm

To investigate the molecular mechanism that AKT3-174aa involved in, we conducted RNA-seq analysis of U251 and U373 cells that were stably expressing circ-AKT3 or circ-AKT3 IRES-mut plasmid. A total of 1873 and 2471 Differentially expressed genes (DEGs) were detected for U251 and U373, respectively (Additional file 2: Figure S3 and S4, Additional file 3: Table S1 and Additional file 4: Table S2). Both DEGs and KEGG (Kyoto Encyclopedia of Genes and Genomes) analysis displayed a notable association with cancer-relation pathways, including PI3K-Akt signaling pathway, TNF signaling pathway, MAPK signaling pathway et al. (Fig. 5a).

As AKT3-174aa shared same sequences as AKT3, we first checked the activation of AKT isoforms in AKT3-174aa overexpression or knockdown cells. AKT3-174aa did not affect the total AKT1, AKT2 or AKT3 mRNA or protein expression level (Fig. 5b). Instead, AKT3-174aa inhibited AKT-thr308 but did not inhibit AKT-ser473 or the phosphorylation of U251, U373, SW1783 and Hs683 cells (Fig. 5b). In human GBM, AKT activation is often associated with PTEN alternation, such as the mutation/loss of heterozygosity or the increase in EGFR mutation/amplification [22]. In AKT3-174aa overexpressed U251 and U373 cells and in AKT-174aa stable knockdown SW1783 and Hs683 cells, we did not find a change in p-EGFR (thr1068). In PTEN wild-type SW1783 and Hs683 cells, PTEN expression was also not affected after AKT3-174aa knockdown (Fig. 5b). Together with the fact that AKT3-174aa attenuated p-AKT-thr308 in PTEN-deficient U251 and U373 cells, we implied that AKT3-174aa exerted its function independently from PTEN or EGFR. AKT3 was reported to have a low expression in GBM tissues but exhibited higher kinase activity than that in the control tissues [11]. To explore whether AKT3-174aa had AKT isoform-specific functions, we used U251 cells with AKT3-174aa overexpression to perform the AKT-isoform specific immunoprecipitation (IP). The overexpression of AKT3-174aa reduced both the AKT2 and AKT3 thr-308 phosphorylation but not the ser-473 phosphorylation, indicating that AKT3-174aa affected more than AKT3. AKT1 phosphorylation was neglectable in U251 cells, as was reported previously [11]. Similar results were acquired in SW1783 cells with the stable knockdown of circ-AKT3 (Fig. 5c). Taking these information together, we showed that AKT3-174aa exerted its effects upstream of p-AKT instead of affecting the AKT isoforms themselves.

AKT activation required several steps as follows: the PH domain is first recruited by activated PI3K, which is located on the cell membrane, to expose the thr-308 site [23]. Next, activated PDK1 phosphorylated thr-308 of AKT [24], and finally, mTOR sequentially phosphorylated the ser-473 site to fully activate AKT [25]. Although PDK1 or p-PDK1 expression was not affected by AKT3-174aa, we found that p-PDK1 was a potential interacting protein in the Flag-AKT3-174aa immunoprecipitation complex by using mass spectrometry (MS), as shown in Fig. 5d. In 293 T cells, Flag-tagged AKT3-174aa could mutually interact with p-PDK1, suggesting the interaction of AKT3-174aa and p-PDK1 in vivo (Fig. 5e). Although purified GST-tagged AKT3-174aa could not pull-down His-tagged PDK1, the in vitro IP by incubation of eukaryotic-purified His-PDK1 with prokaryotic-purified GST-AKT3-174aa could induce their mutual interaction, and this further confirmed that AKT3-174aa interacted with activated PDK1 (Fig. 5f). Immunofluorescence staining in U251 cells transfected with Flag-tagged AKT3-174aa also supported the colocalization of AKT3-174aa with p-PDK1, suggesting that AKT3-174aa preferred to inhibit thr-308 of AKT via interacting with p-PDK1 (Fig. 5g).

### AKT3-174aa is a natural dominant-negative variant of AKT

**Fig. 6:**
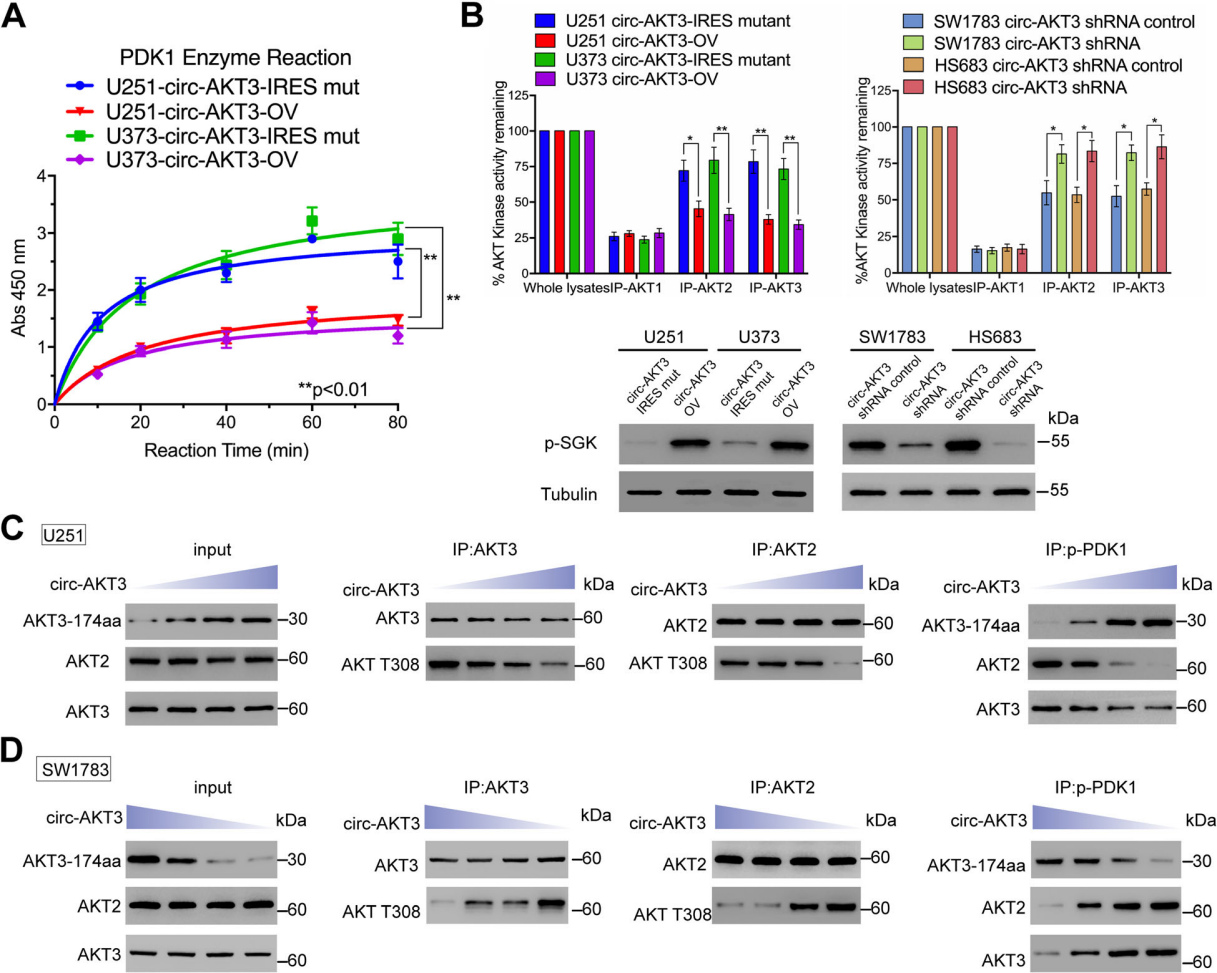
AKT3-174aa competitively interacts with p-PDK1 form ATK2/3. **a** PDK1 kinase activity was determined in U251 and U373 cells with circ-AKT3 overexpression and their control cells at the indicated time point. Error bars represent three independent experiments, **, *p* < 0.01. **b** Upper, AKT kinase activity was determined in U251 and U373 cells with circ-AKT3 overexpression and their control cells (48 h); or in SW1783 and Hs683 cells with circ-AKT3 knocking down and their control cells (48 h). Error bars represent three independent experiments, *, *p* < 0.05, **, *p* < 0.01. Lower, p-SGK level was determined in indicated cells. **c** U251 cells were transfected with increasingly dose of circ-AKT3. IP was performed by using AKT2, AKT3 and p-PDK1 antibodies and followed by immunoblot using indicated antibodies. **d** SW1783 cells were transfected with increasingly dose of circ-AKT3 shRNA. IP was performed by using AKT2, AKT3 and p-PDK1 antibodies and followed by immunoblot using indicated antibodies

Because AKT3-174aa shares the same sequences with AKT3 from amino acids 62–232, we inferred that AKT3-174aa may compete with AKT isoforms to bind to p-PDK1. We showed that the p-PDK1 activity was inhibited after AKT3-174aa overexpression (Fig. 6a). Additionally, AKT kinase activity was inhibited in U251 and U373 AKT3-174aa overexpressed cells, while this activity was enhanced in SW1783 and Hs683 AKT3-174aa stable knockdown cells (Fig. 6b). Furthermore, other p-PDK1 downstream effectors, such as p-SGK [26], were also inhibited significantly by AKT3-174aa (Fig. 6b). Next, we dose-dependently transfected circ-AKT3 into U251 cells and performed immunoprecipitation with AKT2, AKT3 and p-PDK1. With the increased expression of AKT3-174aa, p-AKT-thr308 decreased in both the AKT2 and AKT3 IPs (Fig. 6c). In SW1783 cells with a dose-dependent transfection of circ-AKT3 siRNA, AKT3-174aa deprivation enhanced the p-PDK1 and AKT2/3 interaction and AKT-thr308 phosphorylation (Fig. 6d). Specifically, p-PDK1 pull-down increased the level of AKT3-174aa but decreased the levels of AKT2 and AKT3 in U251 cells; this is in contrast to the effects in SW1783 cells, where we showed that AKT3-174aa is a protein decoy that limited the aberrant activation of p-AKT through dominant-negative regulation (Fig. 6c and d).

### AKT3-174aa suppresses GBM tumorigenicity in vivo

**Fig. 7:**
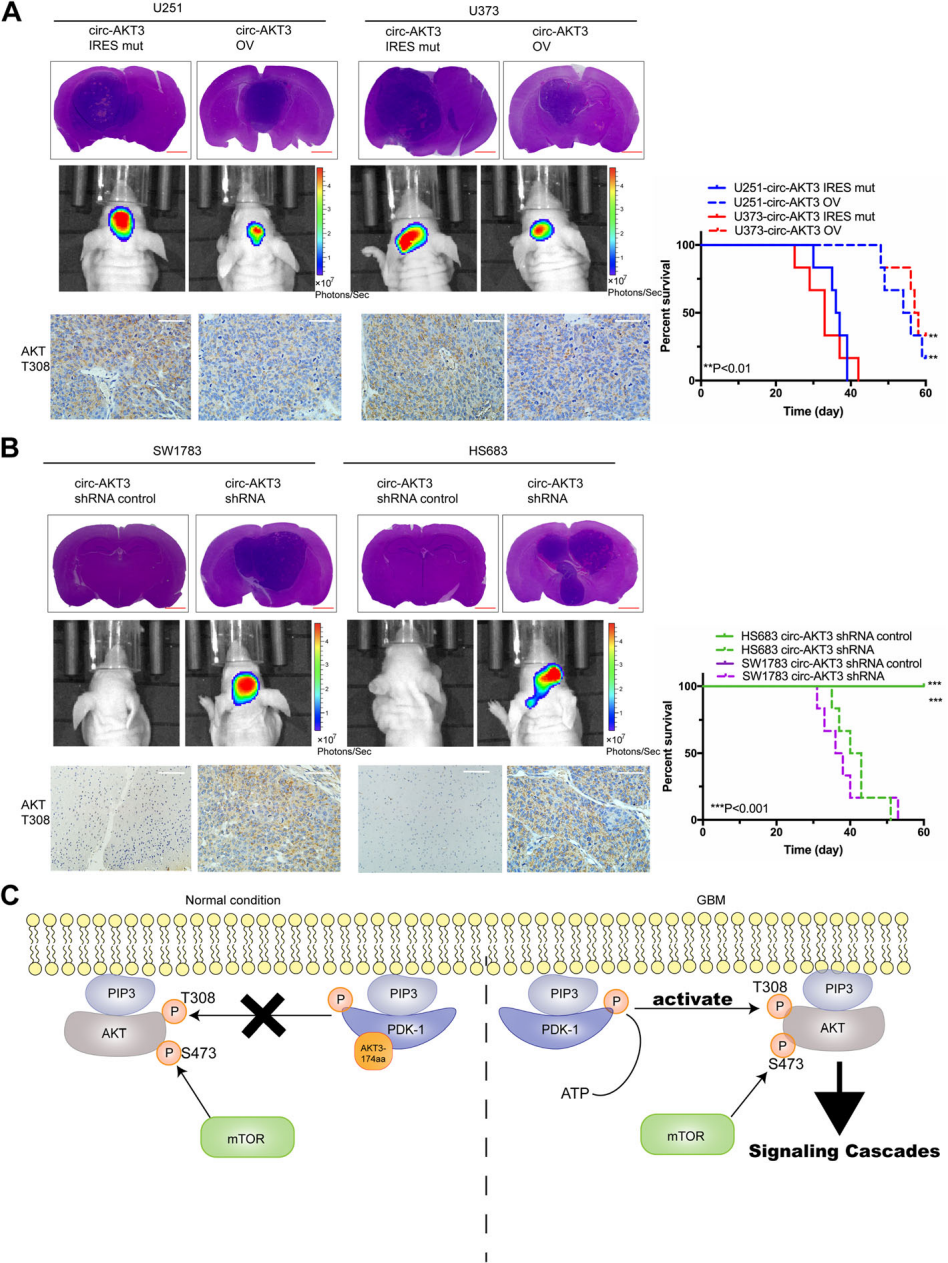
AKT3-174aa negatively correlates the tumorigenicity of GBM cells in vivo*.* **a** U251, U373 cells with circ-AKT3 overexpression and their control cells were intracranially injected into nude mice (1X10^5^ per mice, five mice per group). In vivo tumorigenicity was monitored, and mice were sacrificed when showing clinical symptoms. Mice brain were subjected to HE is staining or IHC staining as indicated. Survival analysis was calculated by Kaplan-Merier curve. *, *p* < 0.05*.* **b** SW1783, Hs683 cells with circ-AKT3 koncking down and their control cells were intracranially injected into nude mice (1X10^5^ per mice, five mice per group). In vivo tumorigenicity was monitored, and mice were sacrificed when showing clinical symptoms. Mice brain were subjected to HE is staining or IHC staining as indicated. Survival analysis was calculated by Kaplan-Merier curve. *, *p* < 0.05*.* **c** Illustration of AKT3-174aa function. Normally, AKT3-174aa interacts p-PDK1 and limits AKT3 thr308 phosphorylation as a molecular decoy. In GBM, loss of AKT3-174aa exposed AKT thr308 to p-PDK1 more easily, promotes AKT activation and sequential signaling cascades

Next, we tested the tumor-suppressive role of AKT3-174aa in mice intracanal tumor models. Nude mice were intracranially injected with AKT3-174aa overexpressed (circular or linearized, respectively) U251 and U373 cells. AKT3-174aa overexpression significantly increased the total survival time of the mouse models, as indicated in Fig. 7a. Conversely, knocking down AKT3-174aa promoted the tumor formation of SW1783 and Hs683 cells in vivo, and the wild-type SW1783 and Hs683 cells could not do the same (Fig. 7b). In mice xenograft tumors, AKT3-174aa overexpression negatively correlated with the expression levels of p-AKT-thr308; this supported the hypothesis that AKT3-174aa inhibits the AKT activation-induced malignant phenotype (Fig. 7a, b). Together, our results show that AKT3-174aa is a critical negative regulator of PI3K/AKT signaling during GBM tumorigenesis (Fig. 7c).

## Discussion

Of the multiple oncogenic signaling pathways that drive GBM tumorigenesis and progression, the RTK/PI3K/AKT pathway plays a central role. Evidence has shown that at least one RTK status was found altered in 67% of GBM cases overall, including EGFR, PDGFRA, MET and FGFR2/3. Meanwhile, the PI3K mutation was found in 25% of GBM cases. Although the PI3K and PTEN mutations were mutually exclusive, 59% of GBM cases showed one or the other. After adding up these genetic alterations, nearly 90% of GBM cases had at least one alteration in RTK/PI3K/AKT signaling and 39% had two or more [3, 27]. AKT represents a nodal point in this pathway. After PI3K activation, AKT was recruited to the plasma through the PH-domain and was them phosphorylated sequentially at thr308 and ser473 to become fully activated [28, 29]. Specifically, PDK1 (another PH domain-containing kinase) directly phosphorylated AKT at thr308, which is the most critical initial step for AKT activation [30]. AKT activation promoted cancer progression through the following three major biological functions: survival, proliferation and growth [31]. So far, three AKT isoforms have been identified (AKT1, AKT 2 and AKT3), which, in general, are broadly expressed, although there are some isoform-specific features. In breast cancer, AKT1 had been demonstrated to suppress while AKT2 promotes migration and invasion [32, 33]. In PTEN-deficient prostate cancer, AKT2, but not AKT1, mediates cellular survival and proliferation [5]. AKT3, but not AKT1 or AKT2, mediated the resistance to apoptosis in BRAF-targeted melanoma cells; promoted anchorage-independent growth in triple negative breast cancer; and controlled the VEGF-induced angiogenesis in ovarian cancer [34–36]. However, the functions of AKT3 in GBM remain controversial: although reports have shown that AKT3 had lower expression levels, its kinase activity was higher than that of AKT2 [11]. Additionally, AKT3 was reported to exert a tumor suppressive function in GBM [12]. In current study, we showed that AKT3 was downregulated in GBM compare with normal brain tissue, which was consistent with the results of previous studies. But the consequences of the *AKT3* downregulation in GBM may not be only due to its higher kinase activity; as AKT3-174aa may also play its part.

We and other groups have shown that circRNAs could encode functional peptides or proteins [13, 14, 37, 38]. Compared with their linear host gene products, circRNA encoded proteins or peptides are usually exerted independently of the biological functions. For example, we found that circPINTexon2 encoded PINT87aa, which is involved in translation elongation, while LINC-PINT showed a functional dependence with PRC2 [14, 39]. The most recent study also supported that circRNAs and linear mRNAs may have individual functions during prostate cancer carcinogenesis [40]. On the other hand, some translational products of the circRNAs involved in their host gene functions, such as SHPRH-146aa protects SHPRH from degradation [14] and FBXW7-185aa induce c-Myc degradation [13]. Interestingly, two recent reports have shown that AKT hyperactivation required SETDB1-induced methylation during tumorigenesis [41, 42]. The reported K64, K140 and K142 methylation sites were all located inside AKT3-174aa, implying that AKT3-174aa also may protect AKT from being methylated; however, further evidence is needed. The potential multiple ‘safe-guard’ role of AKT3-174aa showed that it may be a critical negative regulator of PI3K/AKT signaling expect for PTEN. Vo et al. recently reported that although circRNAs were globally expressed in cancers at low levels, circ-AKT3 seemed to be one of the exclusions [43]. However, their study did not include GBM samples, which were reported to have lower linear AKT3 levels compare with those of the other malignancies including breast cancer, ovarian cancer and melanoma [11, 34–36, 44].

Except for inducing glioma invasion and anchorage-independent growth, AKT3 was reported to significantly activate DNA repair and resistance to radiation and chemotherapy in GBM cases [45]. However, AKT3 did not show any prognostic correlations with GBM patients in the TCGA database. We inferred that the lower expression of AKT3 in some GBM cases may confound the oncogenic characters of AKT3 and may induce the nonsignificant prognostic results. Instead, AKT3-174aa expression showed as a positive correlation with the patients’ total survival with GBM in our study. Considering that AKT3-174aa is one of the few negative regulators of PI3K/AKT signaling (others include PTEN, PHLPP [46], and CTMP [47]), its expression level should be more intensively tested in larger cohort to confirm whether it is an independent biomarker for GBM or other types of human cancers.

Given the central role of PI3K/AKT signaling in GBM, targeting PI3K or AKT is a logical rationale in developing novel therapeutic strategies. However, PI3K inhibitors, such as LY294002/wortmannin, and AKT inhibitors, such as perifosine, were only applied in experimental studies instead of in clinical trials. Basically, the unsatisfactory results of these novel drugs were attributed to the difficulties to penetrate the blood brain barrier and the fast restoration of p-AKT [44]. Although the tumor-suppressive role of AKT3-174aa prevents its druggable potential, we think that the low expression of AKT3-174aa may allow for AKT be easily exposed to p-PDK1 or SETDB1 in GBM, and this makes AKT more sensitive to activation cascades. Thus, effectively restore AKT3-174aa expression may benefit certain GBM patients to PI3K/Akt signaling target therapy, although the optimized delivery system for BBB penetration is required. Our results provide some evidence that the internal balance of the abovementioned genes’ alternative splicing products needs to be restored during GBM target-therapy, which could enhance or maintain the drug efficiency.

## Conclusion

Circ-AKT3 is a newly identified *AKT3* transcriptional variant. Circ-AKT3 encodes AKT3-174aa, which interacts with p-PDK1 as a molecular decoy. Circ-AKT3 and AKT3-174aa are expressed at low levels in GBM, which could more easily induce AKT-thr308 phosphorylation and sequential activation by p-PDK1. Restoring the expression of AKT3-174aa inhibits the cells proliferation, survival and tumorigenicity of GBM in vitro and in vivo. As a novel negative regulator of PI3K/AKT signaling, AKT3-174aa is a potential prognostic marker for GBM patients and may have future potential clinical uses.

## Additional files

Additional file 1: Materials and Methods. (DOCX 34 kb)

Additional file 2: **Figure S1.** A. In the same blot as Fig. 2d, expression of AKT1, AKT2 and AKT T308 level were detected in established cell lines. Loading control was showed in Fig. 2d. B. Left, in the same blot as Fig. 2d, expression of AKT1, AKT2 and AKT T308 level were detected in several GBM samples and paired adjacent normal tissues. C. Expression of AKT3, AKT3-174aa, AKT1, AKT2 and AKT T308 level were detected in several GBM samples and paired adjacent normal tissues. **Figure S2.** Left, Hs683, SW1783 cells were stably overexpressed with control shRNA and circ-AKT3 shRNA. Circ-AKT3 level was detected by PCR. Right, U251 and U373 cells were stably overexpressed with circ-AKT3 IRES mut plasmid, circ-AKT3 plasmid or AKT3-174aa ORF. Circ-AKT3 level was detected by PCR. Error bars represent three independent experiments, **, *p* < 0.01, ***, *p* < 0.001. **Figure S3.** A total of 1873 and 2471 Differentially expressed genes (DEGs) were detected for U251 and U373, respectively. **Figure S4.** The volcano plot(a) and Heatmap (b) of differential expression genes (Fold change > 2 and FDR < 0.05) from ribosomal RNAs depleted RNA-seq. (DOCX 616 kb)

Additional file 3: KEGG pathway analysis of U251 and U373 cells with stable circ-AKT3 overexpression. (XLSX 108 kb)

Additional file 4: Differentally changed genges of U251 and U373 cells with stable circ-AKT3 overexpression. (XLSX 10005 kb)

## Data Availability

Raw sequencing and processed RNA Seq data from this study have been deposited into NCBI SRA: SRP095744 and SRP216548.

## Abbreviations

Circ-AKT3: Circular AKT3
circRNA: circular RNA
EGFR: Epidermal growth factor receptor
GBM: Glioblastoma
IRES: Internal ribosomal entry site
ORF: Open reading frame
PDK1: Phosphoinositide-dependent kinase-1
PI3K: Phosphoinositide 3-kinase
PTEN: Phosphate and tension homology deleted on chromosome ten
RTK: Receptor tyrosine kinase
