# Correction to: A novel tumor suppressor protein encoded by circular AKT3 RNA inhibits glioblastoma tumorigenicity by competing with active phosphoinositide-dependent Kinase-1

**DOI:** 10.1186/s12943-019-1083-2

**Published:** 2019-10-29

**Authors:** Xin Xia, Xixi Li, Fanying Li, Xujia Wu, Maolei Zhang, Huangkai Zhou, Nunu Huang, Xuesong Yang, Feizhe Xiao, Dawei Liu, Lixuan Yang, Nu Zhang

**Affiliations:** 1 Department of Neurosurgery, The First Affiliated Hospital of Sun Yat-sen University, No 58, Zhongshan 2 Road, Guangzhou, Guangdong Province 510080 People’s Republic of China; 2 Guangdong Provincial Key Laboratory of Brain Function and Disease, Precise Medicine Institute, The First Affiliated Hospital of Sun Yat-sen University, Guangzhou, Guangdong 510080 People’s Republic of China; 3 Department of Scientific Research Section, The First Affiliated Hospital of Sun Yat-sen University, Guangzhou, Guangdong Province 510080 People’s Republic of China; 4 Department of Pathology, The First Affiliated Hospital of Sun Yat-sen University, Guangzhou, Guangdong Province 510080 People’s Republic of China

**Correction to: Mol Cancer (2019) 18:131**

**https://doi.org/10.1186/s12943-019-1056-5**

In the published article [1], an error was noticed in Fig. 6b. The western blot results were reversed between the overexpression group and the knockdown group of circ-AKT3. The corrected and updated Fig. 6 is provided below. This error does not affect the findings or conclusions of the article.

**Fig. 6:**
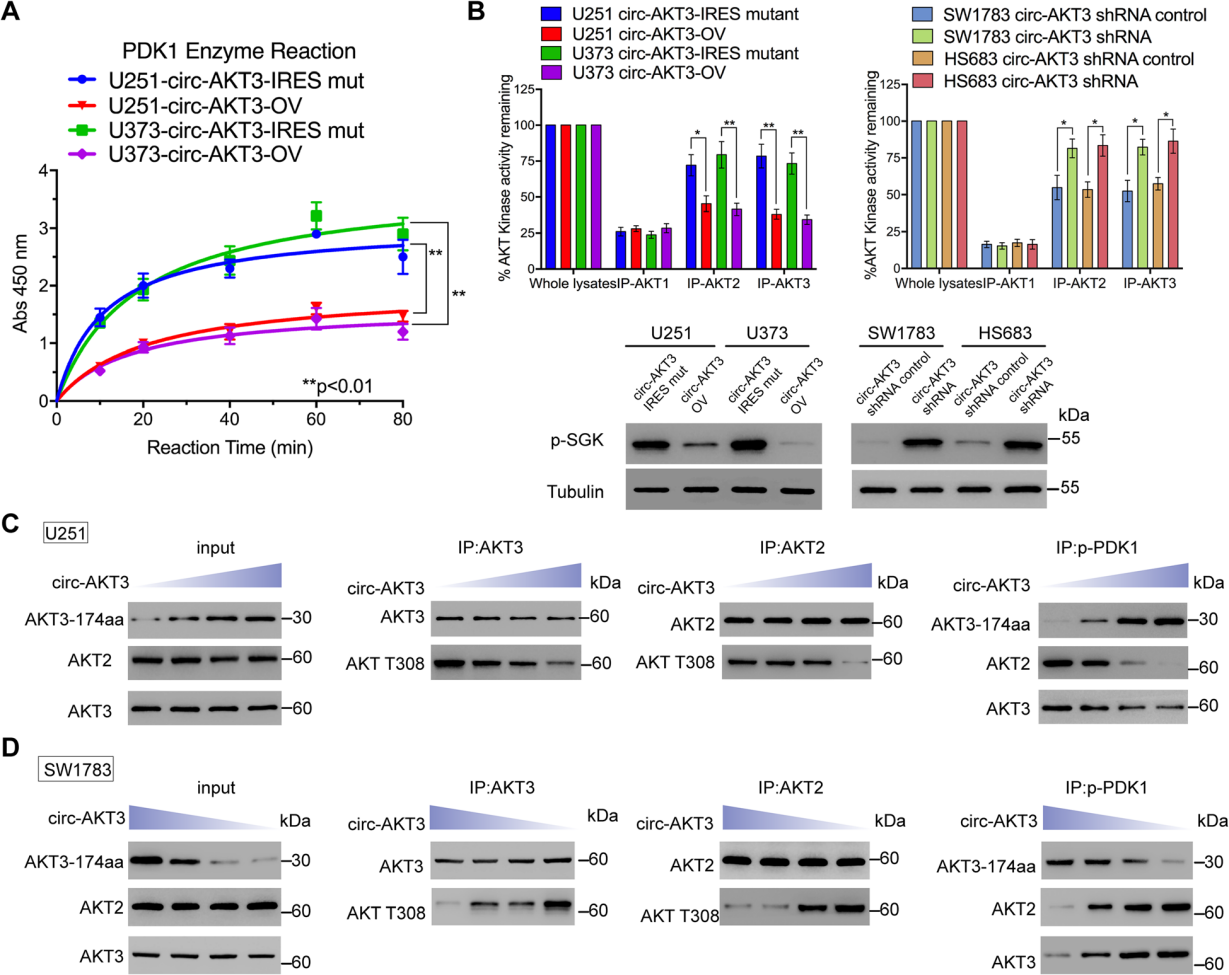
AKT3-174aa competitively interacts with p-PDK1 form ATK2/3. **a** PDK1 kinase activity was determined in U251 and U373 cells with circ-AKT3 overexpression and their control cells at the indicated time point. Error bars represent three independent experiments, **, *p* < 0.01. **b** Upper, AKT kinase activity was determined in U251 and U373 cells with circ-AKT3 overexpression and their control cells (48 h); or in SW1783 and Hs683 cells with circ-AKT3 knocking down and their control cells (48 h). Error bars represent three independent experiments, *, *p* < 0.05, **, *p* < 0.01. Lower, p-SGK level was determined in indicated cells. **c** U251 cells were transfected with increasingly dose of circ-AKT3. IP was performed by using AKT2, AKT3 and p-PDK1 antibodies and followed by immunoblot using indicated antibodies. **d** SW1783 cells were transfected with increasingly dose of circ-AKT3 shRNA. IP was performed by using AKT2, AKT3 and p-PDK1 antibodies and followed by immunoblot using indicated antibodies
