# Correction: A novel tumor suppressor protein encoded by circular AKT3 RNA inhibits glioblastoma tumorigenicity by competing with active phosphoinositide-dependent Kinase-1

**DOI:** 10.1186/s12943-022-01584-y

**Published:** 2022-06-07

**Authors:** Xin Xia, Xixi Li, Fanying Li, Xujia Wu, Maolei Zhang, Huangkai Zhou, Nunu Huang, Xuesong Yang, Feizhe Xiao, Dawei Liu, Lixuan Yang, Nu Zhang

**Affiliations:** 1 Department of Neurosurgery, The First Affiliated Hospital of Sun Yat-sen University, No 58, Zhongshan 2 Road, Guangzhou, Guangdong Province 510080 People’s Republic of China; 2 Guangdong Provincial Key Laboratory of Brain Function and Disease, Precise Medicine Institute, The First Affiliated Hospital of Sun Yat-sen University, Guangzhou, Guangdong 510080 People’s Republic of China; 3 Department of Scientific Research Section, The First Affiliated Hospital of Sun Yat-sen University, Guangzhou, Guangdong Province 510080 People’s Republic of China; 4 Department of Pathology, The First Affiliated Hospital of Sun Yat-sen University, Guangzhou, Guangdong Province 510080 People’s Republic of China; 5 Department of Neurosurgery, The First Affiliated Hospital of Sun Yat-sen University, No 58, Zhongshan 2 Road, Guangzhou, Guangdong Province 510080 People’s Republic of China; 6 Guangdong Provincial Key Laboratory of Brain Function and Disease, Precise Medicine Institute, The First Affiliated Hospital of Sun Yat-sen University, Guangzhou, Guangdong 510080 People’s Republic of China

**Correction to: Mol Cancer 18, 131 (2019)**

**https://doi.org/10.1186/s12943-019-1056-5**

Following publication of the original article [1], errors were identified in the images presented in Figs. 3 and 4; specifically:

- Fig. 3C Representative images showing plate colony formations from different cell lines were incorrectly selected during figure assembly; this affects circ-AKT3 IRES mut U373 (bottom left in left-hand colony formations), and circ-AKT3 shRNA control SW1783 and HS683 (top and bottom left in right-hand colony formations). The correct representative images are now used
- Fig. 4G Representative images of the control group for Empty Vector U373 (bottom left cell line) was incorrectly used. The correct representative image is now used

The corrected figures are provided here. The correction does not have any effect on the results or conclusions of the paper. The original article has been corrected.




