# Supplementary material for: A novel tumor suppressor protein encoded by circular AKT3 RNA inhibits glioblastoma tumorigenicity by competing with active phosphoinositide-dependent Kinase-1
Source: Mol Cancer. 2019 Aug 30;18:131. doi: 10.1186/s12943-019-1056-5 (PMC6716823; doi:10.1186/s12943-019-1056-5)
Supplement: Supplementary file 1 — Materials and Methods. (DOCX 34 kb) [file 12943_2019_1056_MOESM1_ESM.docx]

**Full Materials and Methods**

***RNA-seq analysis and Annotation of circRNAs***

Ten human glioblastoma samples were randomized as the tumor group (TX) and their peripheral normal tissues were randomized as the control group (PX). Total RNAs were treated with RNase-R to eliminate the linear RNAs and purified using RNeasy MinElute Cleanup Kit (Qiagen). Strand-specific library was constructed by using a VAHTS Total RNA-seq (H/M/R) Library Prep Kit from Illumina according to the manufacturer’s instructions. Ribosome RNAs were removed. CircRNAs were fragmented using a fragmentation buffer and reverse transcribed into cDNA with random primers. Second-strand cDNA was synthesized using DNA polymerase I, RNase H, dNTP (dUTP instead of dTTP) and buffer. The cDNA fragments were purified with VAHTSTM DNA Clean Beads, end repaired, supplemented with poly(A), and ligated to Illumina sequencing adapters. Uracil-N-glycosylase (UNG) was used to digest the second-strand cDNA. The digested products were purified with VAHTSTM DNA Clean Beads, PCR amplified, and sequenced using Illumina HiSeqTM 2500 by Gene Denovo Biotechnology Co. (Guangzhou, China).

RNA-seq data were deposited in the SRA database [PRJNA355185 (SRP095744)]. The sequencing results were analyzed as previously described[1]. Briefly, the short reads alignment tool Bowtie2 was used for mapping reads to the ribosome RNA (rRNA) database. The rRNA mapped reads were removed. The remaining reads were further used in alignment and analysis. The removed rRNA reads of each sample were then mapped to a reference genome byTopHat2 (version 2.0.3.12). The reads that could be mapped to the genome were discarded, and the unmapped reads were then collected for circRNAs identifification.20mers from both ends of the unmapped reads were extracted and aligned to the reference genome to identify unique anchor positions within the splice site. Anchor reads that aligned in the reverse orientation (head-to tail) indicated circRNA splicing and were then subjected to fifind_circ to identify circRNAs. The anchor alignments were then extended such that the complete read aligns and the breakpoints were flanked by GU/AG splice sites. A candidate circRNA was called if it was supported by at least two unique back spliced reads in at least one sample. circRNAs were blasted in the circBase for annotation. Those sequences that could not be annotated were defined as novel circRNAs. To quantify circRNAs, back-spliced junction reads were scaled to reads per million mapped reads (RPM), and the formula is shown as follows:

RPM=$\frac{10^6C}{N}$

In this formula, *C* is the number of back-spliced junction reads that uniquely aligned to a circRNA. N is the total number of back-spliced junction reads. To identify differentially expressed circRNAs across samples or groups, the edgeR package (http://www.r-project.org/) was used. We identifified circRNAs with a fold change ≥ 2 and a P value< 0.05 in a comparison between samples or groups as signifificantly differentially expressed circRNAs.

***Northern Blot***

Approximately 10 µg of total RNA was separated in a 1.2% agarose gel containing formaldehyde. The RNA was then transferred to Amersham hybond-N1 membranes (GE Healthcare, Little Chalfont, Buckinghamshire, UK). The membranes were hybridized with digoxin-labeled DNA oligonucleotides specific to circ-AKT3 (listed in Supplementary Table 1) in Church buffer (0.5 M NaPO4, 7% SDS, 1 mM EDTA, 1% BSA, pH 7.5) at 37 °C and washed in 2× SSC (300 mM NaCl, 30 mM Na-citrate, pH 7.0) with 0.1% SDS at room temperature. The membranes were finally exposed on phosphorimager screens and analyzed using Quantity One or Image Lab software (Bio-Rad, Hercules, CA, USA).

***Actinomycin D assay***

HEK293T cells were equally seeded in 5 wells in 24-well plates (5 × 10^4^ cells per well). 24 hours later, the cells were exposed to actinomycin D (2µg/ml, HY-17559, Med Chem Express, Monmouth Junction, NJ, USA) for 0h, 4h, 8h, 12h and 24h, respectively. After that, the cells were harvested and the relative RNA levels of circ-AKT3 and linear-AKT3 were analyzed by qRT-PCR and normalized to the values measured in the mock treatment group (the 0h group).

***RNA fluorescence in situ hybridization (FISH).***

Fluorescence labeled oligonucleotide probes complementary to circ-AKT3 were designed using the Clone Manager suite of analysis tools (Sci Ed Central, listed in Supplementary Table 1). 1×10^4^ Cells were seeded on a coverglass-bottom confocal dish and cultured overnight. RNA FISH assay was performed using RNA FISH kit (Suzhou GenePharma Co, Ltd, Suzhou, China) according to manufacturer’s instruction. Nuclei were stained with 4,6-diamidino-2-phenylindole. Images were acquired on ZEISS LSM 880 with Airyscan (Carl Zeiss Microscopy GmbH, Jena, Germany).

***Plasmids and transfection***

Circ-AKT3 expression plasmid was generated by chemical gene synthesis the sequence of exon3-7 of AKT3, additional circulation promoter sequence and AG/GT splicing sequence were added to the 83bp upstream and 53bp downstream. The linear AKT3-174aa-flag overexpression vector was cloned as a positive control. The Circular RNA expression frame sequence and Linear AKT3-174aa-flag were cloned into the psin-EF2 vector (Daen Gene Co, Ltd, Guangzhou, China) at the EcoRI and BamHI sites. The renilla luciferase (Rluc) and the firefly luciferase (Luc) sequences were amplified from a psicheck2 vector. The Rluc was placed in front and the Luc was placed in the back. The full-length sequences of Rluc-Luc were obtained by overlapping PCR and the flank sequences were connected to pCDNA3.1(+) vector by two restriction enzyme sites NheI and XhoI. The potential IRES sequences of circ-AKT3 were amplified and inserted in the middle of Rluc and Luc by two restriction enzyme sites kpnI and EcoRI introduced by primers.The plasmids were transfected with Lipofectamine 3000 (Invitrogen, Carlsbad, CA, USA) according to the manufacturer’s instructions.

***Stable cell line generation***

Lentiviral vectors expressing were co-transfected with packaging vectors psPAX2 and pMD2G (Addgene) into HEK293T cells for lentivirus production using Lipofectamine 2000 in accordance to the manufacturer’s instructions. To establish stable cell lines, the cell lines were lentivirus infected and selected with 2 μg/ml puromycin for 72 h. To generate circ-AKT3 stable knockdown SW1783 and HS683 cell lines, lentiviral-induced shRNA (GenePharma, Shanghai, China) was used according to the manufacturer’s instructions (shRNA sequences are listed in Supplementary Table 1).

***Western blotting***

After extraction with RIPA buffer with protease inhibitor and phosphatase inhibitor cocktails (Pierce Biotechnology, Rockford, IL, USA) and quantified with a BCA kit (Thermo Fisher Scientific, Waltham, MA, USA), equal loading proteins of cell lysates or tissue lysates were added to each well of SDS PAGE. Followed by electrophoresising, transfer-membraning, and blocking with 5% non-fat milk in PBST for 1 h, then diluted primary antibodies were incubated at 4°C overnight. After washing with PBST every 10 min for 3 times, diluted horseradish peroxidase (HRP)-conjugated secondary antibodies (# 31430，# 31460，Invitrogen, Carlsbad, CA, USA) were incubated for 1h at room temperature, then the signals were visualized.

Antibodies against pan-AKT (#4691, 1:1000), phospho-AKT Thr308 (#13038, 1:1000), phospho-AKT Ser473 (#4060, 1:1000), AKT1 (#2938, 1:1000), AKT2 (#3063, 1:1000), AKT3 (#14982, 1:1000), and p-PDK1 (#3438, 1:1000) were from Cell Signaling Technology (Danvers, MA, USA). Antibodies against γ- H2AX (ab2893; 1:1000), PTEN (ab32199; 1:10000), EGFR (ab32430; 1:5000) were from Abcam (Cambridge, MA, USA). Antibodies against flag (F1804, 1 mg/mL; 1:1000) and beta Tubulin (T5201; 1:5000) were from sigma-aldrich (St. Louis, MO, USA).

***Reverse transcription and real-time PCR***

Reverse transcription for mRNA and circRNAs was performed with an MMLV-RT kit (TaKaRa, Tokyo, Japan) using random hexamers according to the manufacturer’s instructions. PCR was subsequently performed with a 1:10 dilution of reverse-transcribed cDNA. The PCR product was run in a 2% agarose gel. Real-time quantitative polymerase chain reaction (RT-qPCR) was performed using an Applied Biosystems PCR System (ABI 7500). RT-qPCR SYBR Green Mix (TaKaRa, Tokyo, Japan) was employed with 50 nM of forward and reverse primers in a 20 μl reaction system. The primer sequences for the analyzed genes are summarized in Supplementary Table 1. The relative expression levels were calculated according to 2-ΔΔCT. To determine the absolute quantity of RNA, the purified PCR product amplified from cDNA corresponding to the circ-AKT3 sequence was serially diluted to generate a standard curve.

***Immunoprecipitation (IP)***

Cells were lysed in co-IP buffer (10 mM HEPES [pH 8.0], 300 mM NaCl, 0.1 mM EDTA, 20% glycerol, 0.2% NP-40, protease and phosphatase inhibitors). The lysates were then centrifuged and cleared via incubation with 25 μl protein A/G agarose (GIBCO BRL, Grand Island, NY, USA) for 1.5 h at 4°C. The pre-cleared supernatant was subjected to IP using the indicated primary antibodies at 4 °C overnight. Then, the protein complexes were collected via incubation with 30 μl protein A/G gel for 2 h at 4 °C. The collected protein complexes were separated via SDS-PAGE and analyzed by performing MS or blotting.

***AKT kinase activity and PDK kinase activity***

Cells were lysed in co-IP buffer and non-denatured AKT1, AKT2, AKT3 or PDK1 proteins for kinase assay were obtained using Catch and Release® v2.0 Reversible Immunoprecipitation System (Millipore, Burlington, MA, USA) according to manufacturers’ instruction. In brief, 500ug whole-cell lysates, antibodies against different AKT isoforms and PDK1 (AKT1, #2938; AKT2, #3063; AKT3, #14982; Cell Signaling Technology, Danvers, MA, USA; PDK1, #10026-1-AP, Proteintech Group, Inc, Rosemont, IL, USA) and 10 mL of antibody capture affinity ligand were mixed and placed in a Catch and Release v2.0 spin column containing 0.5 mL of prepacked immunoprecipitation (IP) capture resin. After 12 hours’ end-over-end shaking, the column was centrifuged, washed and then eluted with non-denaturing elution buffer. The IP-Akt1, IP-Akt2, and IP-Akt3 elution were monitored for AKT kinase activity respectively using AKT/PKB kinase assay screening kit (Medical & Biological Laboratories Co, Ltd, Naka-ku, Nagoya, Japan). For PDK1 kinase activity detection, IP-PDK1 was subjected to test using PDK1 Kinase Assay/Inhibitor Screening Kit (Medical & Biological Laboratories Co, Ltd, Naka-ku, Nagoya, Japan) according to manufactures’ instruction.

***Cell proliferation assays.***

Cells were seeded into 96-well plates. At the indicated time points, the cells were incubated with 100ul medium containing 10% WST-8 regent for 2h at 37℃using Cell Counting Kit-8 (Dojindo, Kumamoto, Japan). The absorbance was measured at 450 nm. All experiments were performed in triplicate.

***EdU incorporation assay.***

Cell proliferation was determined by EdU assay kit (iClick™ EdU Andy Fluor™ 594 Imaging Kit, A005, GeneCopoeia, Rockville, MD, USA). Indicated cells were seeded in coverslips for 24 hours under normal condition and proliferation rate was determined according to the manufacture’s instruction.

***Colony formation assays.***

Cells were plated in 6-well plates (1000 cells per plate), cultured for 10 days, fixed with 10% formaldehyde for 5 min, stained with 1.0% crystal violet for 30 s, and counted. All experiments were performed in three biological replicates.

***Soft-agar growth assay***

Blend a total of 1x10^4 stably transfected cells in prewarmed (37 °C) 0.6% soft-agar containing regular medium and pour the mixture on top of 1.2% agar in a 6-well plate. Add five drops of normal growth media per 3 days. After 2 weeks, the number and size of colonies were counted and measured in ten random fields using ImageJ. All experiments were performed in three biological replicates.

***Cell cycle analyses.***

Cells were harvested via trypsinization, washed in ice-cold PBS, fixed in ice-cold 75% ethanol in PBS, centrifuged at 4 °C and suspended in PBS. RNase A (Epicenter Technologies, Madison, WI, USA) was then added at a final concentration of 4 mg/ml, followed by incubation at 37 °C for 30 min. Then, 20 mg/ml propidium iodide (Beyotime, Shanghai, China) was added, and the sample was incubated for 20 min at room temperature. The cells were finally analyzed via flow cytometry (BD Biosciences, San Jose, CA, USA).

***LC-MS analysis.***

Proteins were separated via SDS-PAGE, and the gel bands were manually cut and digested with sequencing-grade trypsin (Promega, Madison, WI, USA). The digested peptides were analyzed with a QExactive mass spectrometer (Thermo Fisher Scientific, Waltham, MA, USA). The fragment spectra were analyzed using the National Center for Biotechnology Information nonredundant protein database with Mascot (Matrix Science, Boston, MA, USA).

***GST pull-down and in vitro binding assay***

6xHis-tagged and GST-tagged proteins were expressed in E. coli and purified using a Ni-His purification kit (Novagen, Madison, WI, USA) and GST beads, respectively. For the GST pull-down assay, His-PDK1 were mixed with GST-AKT3-174aa for 4 h and then subjected to IP with the indicated primary antibodies at 4 °C overnight. The collected protein complexes were washed 6 times with co-IP buffer and analyzed by western blotting.

Eukaryotic-purified His-PDK1 and prokaryotic-purified GST-AKT3-174aa were incubate for the in vitro IP with the indicated primary antibodies at 4 °C overnight. The collected protein complexes were washed 6 times with co-IP buffer and analyzed by western blotting.

***IF staining and confocal microscopy.***

Cells were grown on chamber slides pre-coated with poly-L-ornithine and fibronectin. The cells were fixed with 4% paraformaldehyde, permeabilized for 5 min with PBS containing 0.1% Triton X-100 (PBS-T), quenched with 50 mM NH_4_Cl in PBS-T, and blocked with 1% BSA in PBS-T. Immunostaining was performed with appropriate primary and secondary antibodies, and images were acquired using ZEISS LSM 880 with Airyscan (Carl Zeiss Microscopy GmbH, Jena, Germany).

***Intracranial injection***

All mouse experiments were approved by the Institutional Animal Care and Use Committee of the Sun Yat-sen University. We intracranially injected 1×10^5^ cells of the indicated cell types into 4-week-old female athymic nude mice (purchased from the Animal center, Sun Yat-sen University). Five mice were injected for each group. The mice were sacrificed after 100 days or when they showed the clinical symptoms such as weight loss and specific neurological signs indicating the presence of braintumors (such as seizures, ataxia and lethargy). The brain of each mouse was harvested, fixed in 4% formaldehyde and embedded in paraffin. Tumor formation and phenotypes were determined through histologic analysis and assessed in hematoxylin and eosin-stained sections. The total survival curves were calculated.

***Hematoxylin and Eosin (HE) Staining***

Paraffin embedded mice brain was sliced (4μM), rehydrated through an xylene and ETOH series, and then stained with Hematoxylin (Gill’s 1X) for 5 minutes. Rinse slides in running tap water for 5 minutes and dunk in Acid Alcohol (1%HCl in 70%ETOH) 2-3 times until the sections turn pink. Rinse slides in tap water again for 3-5 minutes and 5-6 slow dunks in ammonia Water (1mL NH4OH in 1L H2O). Rinse slides 3-5 minutes in tap water and then counterstain with Eosin Y solution for 1 minute. Dehydrate through ETOH series and clear in xylene series. Coverslip slides were mounted in ProLong Gold (Invitrogen, Carlsbad, CA, USA) and left overnight at room temperature.

1. Yang Y, Gao X, Zhang M, Yan S, Sun C, Xiao F, Huang N, Yang X, Zhao K, Zhou H, et al: **Novel Role of FBXW7 Circular RNA in Repressing Glioma Tumorigenesis.** *J Natl Cancer Inst* 2018, **110**.
