# Supplementary material for: A novel tumor suppressor protein encoded by circular AKT3 RNA inhibits glioblastoma tumorigenicity by competing with active phosphoinositide-dependent Kinase-1
Source: Mol Cancer. 2019 Aug 30;18:131. doi: 10.1186/s12943-019-1056-5 (PMC6716823; doi:10.1186/s12943-019-1056-5)
Supplement: Supplementary file 2 — Figure S1. A. In the same blot as Fig. 2d, expression of AKT1, AKT2 and AKT T308 level were detected in established cell lines. Loading control was showed in Fig. 2d. B. Left, in the same blot as Fig. 2d, expression of AKT1, AKT2 and AKT T308 level were detected in several GBM samples and paired adjacent normal tissues. C. Expression of AKT3, AKT3-174aa, AKT1, AKT2 and AKT T308 level were detected in several GBM samples and paired adjacent normal tissues. Figure S2. Left, Hs683, SW1783 cells were stably overexpressed with control shRNA and circ-AKT3 shRNA. Circ-AKT3 level was detected by PCR. Right, U251 and U373 cells were stably overexpressed with circ-AKT3 IRES mut plasmid, circ-AKT3 plasmid or AKT3-174aa ORF. Circ-AKT3 level was detected by PCR. Error bars represent three independent experiments, **, p < 0.01, ***, p < 0.001. Figure S3. A total of 1873 and 2471 Differentially expressed genes (DEGs) were detected for U251 and U373, respectively. Figure S4. The volcano plot(a) and Heatmap (b) of differential expression genes (Fold change > 2 and FDR < 0.05) from ribosomal RNAs depleted RNA-seq. (DOCX 616 kb) [file 12943_2019_1056_MOESM2_ESM.docx]

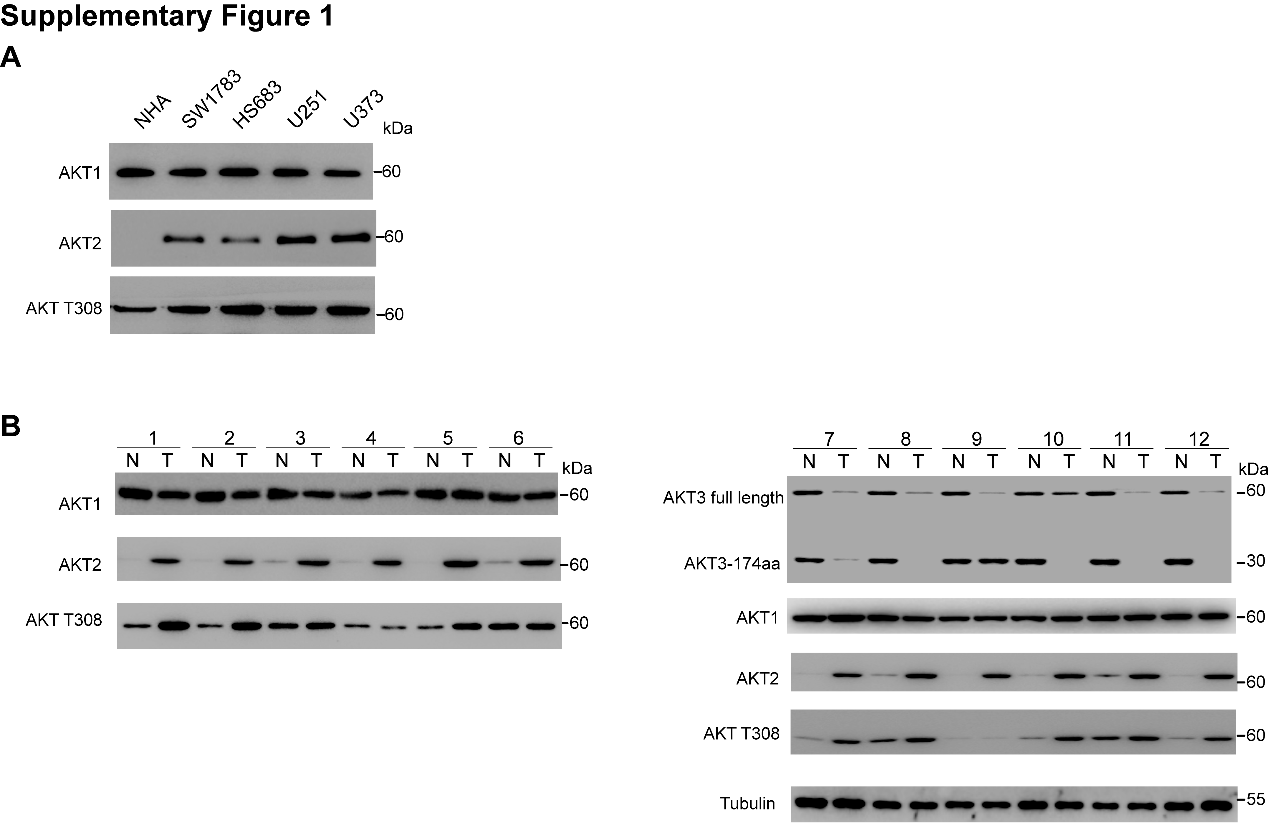


A. In the same blot as Figure 2D, expression of AKT1, AKT2 and AKT T308 level were detected in established cell lines. Loading control was showed in Figure 2D.

B. Left, in the same blot as Figure 2D, expression of AKT1, AKT2 and AKT T308 level were detected in several GBM samples and paired adjacent normal tissues.

C. Expression of AKT3, AKT3-174aa, AKT1, AKT2 and AKT T308 level were detected in several GBM samples and paired adjacent normal tissues.

**
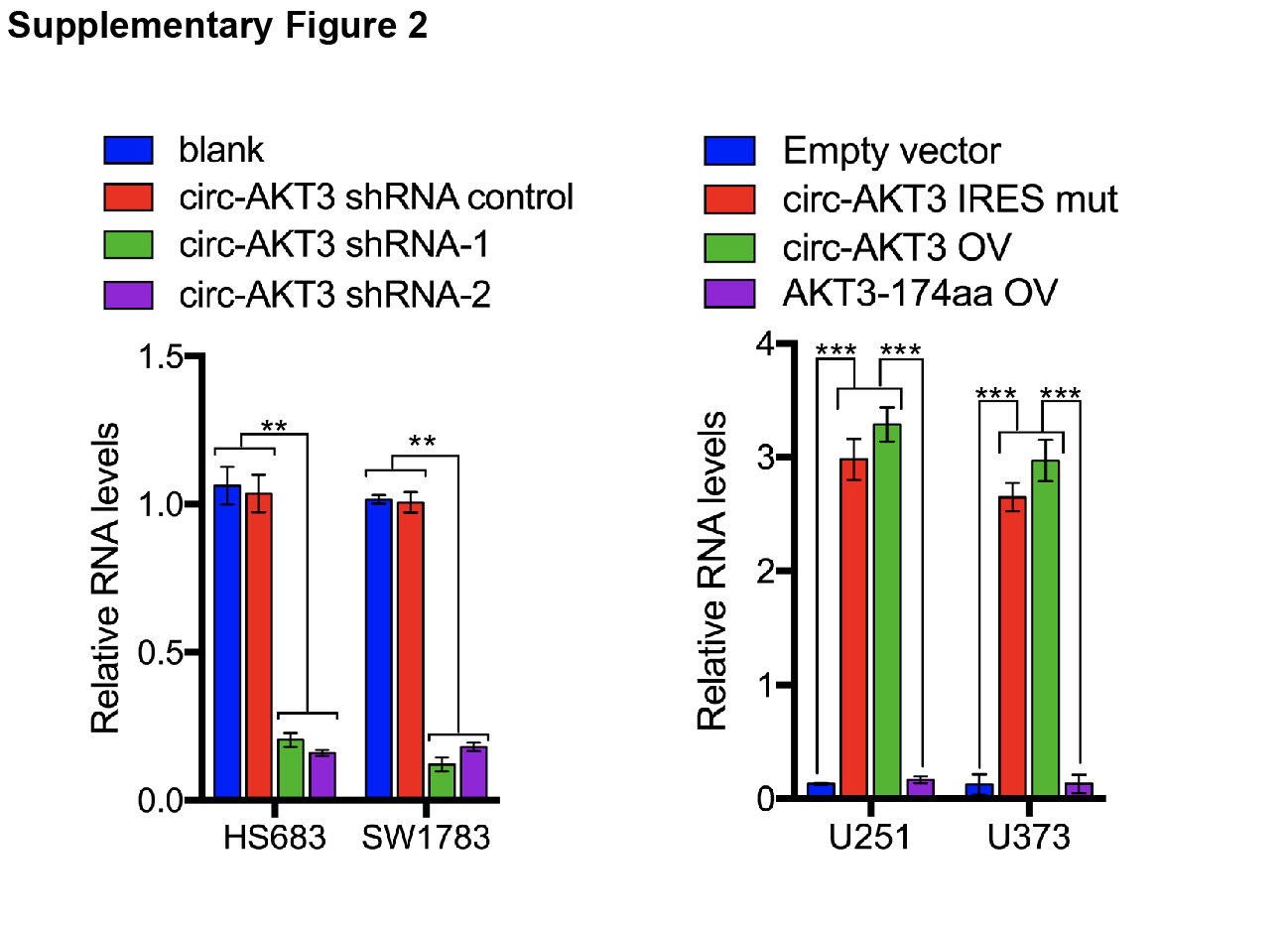
**

Left, Hs683, SW1783 cells were stably overexpressed with control shRNA and circ-AKT3 shRNA. Circ-AKT3 level was detected by PCR. Right, U251 and U373 cells were stably overexpressed with circ-AKT3 IRES mut plasmid, circ-AKT3 plasmid or AKT3-174aa ORF. Circ-AKT3 level was detected by PCR. Error bars represent three independent experiments, **, p<0.01, ***, p<0.001.


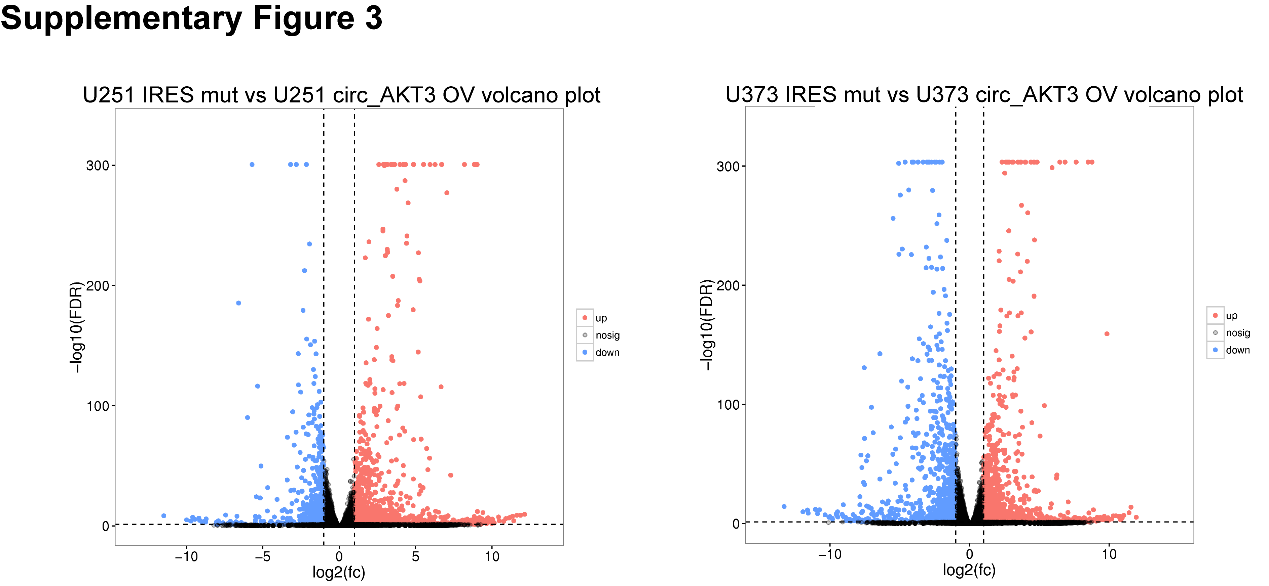


A total of 1,873 and 2,471 Differentially expressed genes (DEGs) were detected for U251 and U373, respectively.

**Supplemental Figure 4**


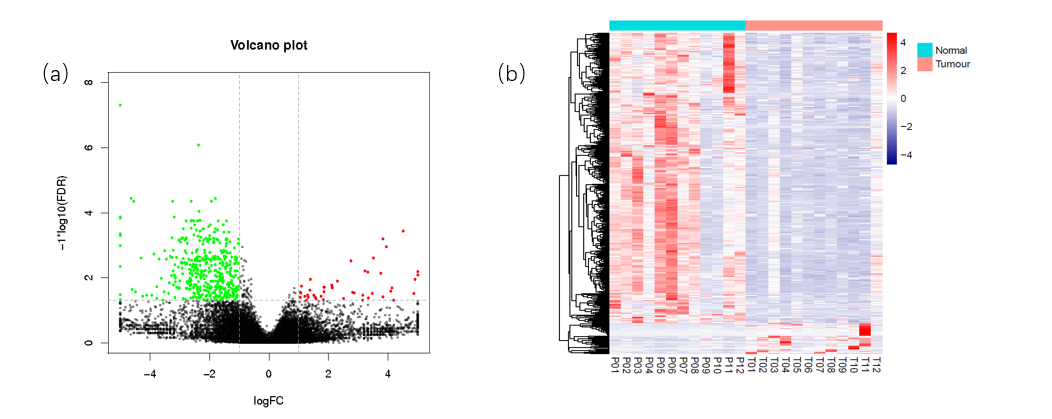


The volcano plot(a) and Heatmap (b) of differential expression genes (Fold change >2 and FDR < 0.05) from ribosomal RNAs depleted RNA-seq.
